# Genomic organization, evolution, and expression of photoprotein and opsin genes in *Mnemiopsis leidyi*: a new view of ctenophore photocytes

**DOI:** 10.1186/1741-7007-10-107

**Published:** 2012-12-21

**Authors:** Christine E Schnitzler, Kevin Pang, Meghan L Powers, Adam M Reitzel, Joseph F Ryan, David Simmons, Takashi Tada, Morgan Park, Jyoti Gupta, Shelise Y Brooks, Robert W Blakesley, Shozo Yokoyama, Steven HD Haddock, Mark Q Martindale, Andreas D Baxevanis

**Affiliations:** 1 Genome Technology Branch, Division of Intramural Research, National Human Genome Research Institute, National Institutes of Health, 50 South Drive, Bethesda, MD 20892, USA; 2 Sars International Centre for Marine Molecular Biology, Thormøhlensgt. 55, N-5008, Bergen Norway; 3 Monterey Bay Aquarium Research Institute, 7700 Sandholdt Road, Moss Landing, CA 95039, USA; 4 Department of Biology, University of North Carolina at Charlotte, 9210 University City Boulevard, Charlotte, NC 28223, USA; 5 Kewalo Marine Laboratory, Pacific Biosciences Research Center, University of Hawaii at Manoa, 41 Ahui Street, Honolulu, HI 96813, USA; 6 Department of Biology, Emory University, 1510 Clifton Road NE, Atlanta, GA 30322, USA; 7 NIH Intramural Sequencing Center, National Human Genome Research Institute, National Institutes of Health, 5625 Fishers Lane, Rockville, MD 20852, USA

**Keywords:** Bioluminescence, ctenophore, Mnemiopsis leidyi, opsin, photocyte, photoprotein, photoreception, phototransduction

## Abstract

**Background:**

Calcium-activated photoproteins are luciferase variants found in photocyte cells of bioluminescent jellyfish (Phylum Cnidaria) and comb jellies (Phylum Ctenophora). The complete genomic sequence from the ctenophore *Mnemiopsis leidyi*, a representative of the earliest branch of animals that emit light, provided an opportunity to examine the genome of an organism that uses this class of luciferase for bioluminescence and to look for genes involved in light reception. To determine when photoprotein genes first arose, we examined the genomic sequence from other early-branching taxa. We combined our genomic survey with gene trees, developmental expression patterns, and functional protein assays of photoproteins and opsins to provide a comprehensive view of light production and light reception in *Mnemiopsis*.

**Results:**

The *Mnemiopsis *genome has 10 full-length photoprotein genes situated within two genomic clusters with high sequence conservation that are maintained due to strong purifying selection and concerted evolution. Photoprotein-like genes were also identified in the genomes of the non-luminescent sponge *Amphimedon queenslandica *and the non-luminescent cnidarian *Nematostella vectensis*, and phylogenomic analysis demonstrated that photoprotein genes arose at the base of all animals. Photoprotein gene expression in *Mnemiopsis *embryos begins during gastrulation in migrating precursors to photocytes and persists throughout development in the canals where photocytes reside. We identified three putative opsin genes in the *Mnemiopsis *genome and show that they do not group with well-known bilaterian opsin subfamilies. Interestingly, photoprotein transcripts are co-expressed with two of the putative opsins in developing photocytes. Opsin expression is also seen in the apical sensory organ. We present evidence that one opsin functions as a photopigment *in vitro*, absorbing light at wavelengths that overlap with peak photoprotein light emission, raising the hypothesis that light production and light reception may be functionally connected in ctenophore photocytes. We also present genomic evidence of a complete ciliary phototransduction cascade in *Mnemiopsis*.

**Conclusions:**

This study elucidates the genomic organization, evolutionary history, and developmental expression of photoprotein and opsin genes in the ctenophore *Mnemiopsis leidyi*, introduces a novel dual role for ctenophore photocytes in both bioluminescence and phototransduction, and raises the possibility that light production and light reception are linked in this early-branching non-bilaterian animal.

## Background

Bioluminescence is observed in a wide variety of organisms across the tree of life. Luminous organisms include bacteria, dinoflagellates, radiolarians, fungi, ctenophores, cnidarians, annelids, mollusks, arthropods, echinoderms, tunicates, and fishes [1]. These organisms utilize bioluminescence for essential functions ranging from defense to reproduction. The ability to produce light always entails a chemiluminescent reaction where the light-emitting substrate - a luciferin - is oxidized by a specific enzyme - a luciferase. Luciferins and luciferases are highly variable in their chemical structure and protein sequence. For this reason, it is thought that bioluminescence arose independently many times throughout evolution [1].

Coelenterazine is the predominant luciferin observed in the ocean environment [1] and is the specific type of luciferin used in the bioluminescence of jellyfish (Phylum Cnidaria) and comb jellies, or ctenophores (Phylum Ctenophora). Because the complete biosynthesis pathway of coelenterazine is not yet known, it is unclear whether ctenophores synthesize coelenterazine or obtain it from external sources, such as through their diet, as is seen with other species [2-4], including hydrozoan cnidarians [5].

Calcium-activated photoproteins are a special class of luciferase found in cnidarians and ctenophores. In these organisms, the factors required for light emission, including the luciferin (coelenterazine) and oxygen, undergo a covalent reaction in which a peroxy intermediate of the coelenterazine is formed. This is bound to the photoprotein as one complex that, in turn, produces light upon binding another cofactor, Ca^2+^. Cells containing photoproteins are capable of emitting light in proportion to the amount of photoprotein complex present within them [6], which is in contrast to a typical luciferin-luciferase reaction, where turnover occurs. In those cases, there may be an excess of luciferase, or one luciferase may catalyze multiple reactions, but the total amount of light emitted is proportional to the amount of luciferin present. Importantly, the term photoprotein can refer to the photoprotein complex or to the luciferase alone (also known as the apo-protein), excluding the substrate and oxygen. It is in this latter sense that we use the terms photoprotein and photoprotein gene from here onwards. The best-known photoproteins are aequorin, from the hydromedusan jellyfish *Aequorea victoria*, and mnemiopsin, from the ctenophore *Mnemiopsis leidyi*, both of which were first purified in the 1960s and 1970s [7-9]. Aequorin was subsequently cloned and sequenced [10,11]. Since then, photoproteins have been cloned from a number of hydromedusan (Phylum Cnidaria) species. These include mitrocomin from *Mitrocoma cellularia *[12], clytin from *Clytia gregarium *[13], and obelin from both *Obelia longissima *and *O. geniculata *[14,15], as well as other photoproteins from *Aequorea *species such as *A. coerulescens, A. macrodactyla*, and *A. parva*. Berovin and bolinopsin from the ctenophores *Beroe abyssicola *[16,17] and *Bolinopsis infundibulum *[18,19] were subsequently cloned and sequenced. Recently, sequences for two photoproteins from *Mnemiopsis leidyi*, named *mnemiopsin 1 *and *mnemiopsin 2*, have been reported [20,21].

Photoproteins are also EF-hand calcium-binding domain proteins related to calmodulin, troponin C, myosin, spectrin, and sarcoplasmic binding protein [22]. EF-hand proteins are distinct from other calcium-binding proteins in that they have calcium-binding helix-loop-helix motifs characterized by a 'canonical' sequence loop region of 12 contiguous residues that provides the oxygen ligands needed for calcium ion coordination [22]. EF-hand domains are usually present in pairs in proteins; this pairing seems to be important for proper protein folding and may increase the affinity of each EF-hand for calcium [22-24]. All photoproteins have three functional EF-hand domains (termed I, III, and IV) that are used to bind calcium. The crystal structures of aequorin and clytin showed that a fourth putative EF-hand domain (II) has the characteristic structural features of an EF-hand motif but not the canonical sequence normally seen within the calcium-binding loop [25]. In addition, its sequence does not conform to EF-hand profiles catalogued in domain databases such as Pfam and SMART.

Biochemical isolations have shown that native photoproteins consist of a mixture of 'isoforms' (also called isoproteins or isospecies) and that there can be differences in properties among isoforms from a single taxon. Aequorin, for example, consists of at least a dozen isoforms that differ in isoelectric point but not in molecular size [26,27]. Mnemiopsin was found to consist of two major isoforms (named mnemiopsin-1 and mnemiopsin-2), each with two or three minor isoforms [9,28]. The different isoforms can have measurable functional differences; for example, the rate constant for decay of light emission in 100 mM Ca^2+ ^was greater for mnemiopsin-2 than for mnemiopsin-1 [28]. Over two decades ago, it was suggested that some of the aequorin isoforms may actually represent different gene products [29]. The sequencing of the *Mnemiopsis *genome offers the first definitive evidence that all of the different photoprotein isoforms are indeed products of different (or separate) individual genes. Furthermore, these data allow for an examination of the genomic organization of these photoprotein genes.

The evolutionary history of the photoprotein gene family has not been systematically characterized to date. Previous studies have explored photoprotein phylogenetic relationships within jellyfish (hydromedusan) species [30], comparing both their sequences and structures [31]. Recently, Aghamaali *et al. *[20] performed sequence comparisons of ctenophore and hydromedusae photoproteins. However, no investigations have gone beyond the hydromedusan and ctenophoran representatives, potentially obscuring the evolutionary history of this gene family by omitting sequences from other phyla. Here, we have combined all publicly available photoprotein sequence data with the new set of *Mnemiopsis *photoprotein sequences generated through our whole-genome sequencing project [32], as well as with photoprotein-like sequences we identified through bioinformatic searches of genomes of additional non-bilaterian taxa, to reconstruct the phylogeny of this gene family. This approach allowed us to determine when the origin of the gene family occurred in relation to the emergence of the metazoa, and, for the first time, to demonstrate its presence in non-bioluminescent organisms.

Nearly all ctenophores are capable of bioluminescence [33], producing flashes of light in light-producing cells (photocytes) upon stimulation in dark conditions. Interestingly, comb plate cilia diffract light to produce a rainbow of colors and give ctenophores their characteristic iridescent appearance [34], which is often mistaken for bioluminescence. The literature regarding bioluminescence in *Mnemiopsis *dates back to at least the early 20^th ^century [35,36]. In *Mnemiopsis*, light production is confined to photocytes, which are associated with the eight meridional canals underlying the longitudinal comb rows and where they extend onto the lobes. Waves of luminescence can propagate in either direction from the point of the stimulation [35,37,38]. The distribution of photocytes within the meridional canals is asymmetric and discontinuous; photocytes are only found on one side of each canal adjacent to the minor body axes, on the same side as the testes, but not on the side containing the ovaries [39]. During gamete differentiation and embryonic development, light production is first detected in eggs [40], and again at a stage during embryonic development that is closely correlated with the initiation of comb plate growth, approximately 8 h post-fertilization (hpf) [38].

One property of ctenophore photoproteins that distinguishes them from hydromedusan photoproteins is that they are photoinhibited upon light exposure [40-42]. This phenomenon is reversible *in vivo *by returning animals to the dark, but photoinhibition of photoprotein extracts cannot be reversed in the same way. The inhibition has been shown to destroy the intermediate photoprotein complex but does not result in the release of oxygen [42]. Several investigators have suggested that the luminescent response to electrical or mechanical stimulation in *Mnemiopsis *involves a nerve net [36,37,43], and that luminescence is neurally controlled [39,44].

In this study, we used a model ctenophore species to investigate the evolutionary history, genomic organization, and developmental expression patterns of the photoproteins - a gene family that represents a special class of luciferases involved in bioluminescence emission. In a similar manner, we explored the *Mnemiopsis *opsins - genes involved in light sensing. By virtue of its early-branching position on the animal tree, *Mnemiopsis *provides a valuable perspective on the evolution and function of gene families and cell types in early animals. Our observation of co-expression of opsin and photoprotein genes in developing ctenophore photocytes led us to hypothesize a dual role for *Mnemiopsis *photocytes in light sensing and light production. Bioluminescence by bacterial symbionts (reviewed by [45]) and host squid gene expression of phototransduction cascade genes [46] have been shown to occur together in the squid light organ. Tong *et al. *[46] hypothesize that the two phenomena are functionally linked in the light organ in that system. Although there is no microbial involvement in the ctenophore luminescence system, our results led us to hypothesize a similar dual role for photocytes in both light production and light sensing in *Mnemiopsis*.

## Results and discussion

### Ten photoproteins are located in two tandemly arrayed clusters in the *Mnemiopsis *genome

**Figure 1:**
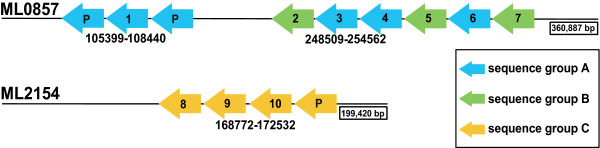
**Genomic arrangement of *Mnemiopsis *photoprotein genes based on the current draft genome assembly**. There are at least 10 putative full-length photoprotein genes, including seven on scaffold ML0857 and three on ML2154. There are also three partial photoprotein genes, indicated by a 'P'. Photoprotein sequences fall into three sequence groups based on similarity. Sequence groups are color-coded and labeled A, B, or C. Genomic coordinates are indicated below each gene cluster. Scaffold lengths (bp) are shown in black boxes at the end of the scaffold. Scaffolds and genes are not drawn to scale.

The availability of the whole genome sequence of *Mnemiopsis *provided us an opportunity to examine the genomic content and context of the luciferases involved in bioluminescence in an early diverging metazoan phylum. This is the first genome available for any species known to utilize calcium-regulated photoproteins for bioluminescence, and, to our knowledge, the first genomic sequence from any bioluminescent animal. We identified 10 photoprotein genes in the *Mnemiopsis *genome organized in two clusters and comprising three sequence similarity groups (A, B, and C; Figure 1). All predicted photoproteins are single exon genes. In some cases, putative photoprotein genes were mispredicted or missed entirely in our original set of predicted gene models. In these cases, we made adjustments based on manual curation and then updated the set of gene models. Rather than adopt the traditional naming scheme for these genes (that is, *mnemiopsin*), we propose to name them *MleiPP1 *through *MleiPP10 *(where 'Mlei' is a combination of the genus and species names, and 'PP' stands for photoprotein). We opted for this naming scheme to clearly convey the function of the protein, as well as to avoid confusion with the names previously used for photoproteins from *Mnemiopsis *- specifically, those used for purified proteins mnemiopsin-1 and -2 [9] and cloned genes *mnemiopsin 1 *and *2 *[21], which may or may not correspond directly with the purified proteins of the same name. This new naming scheme will also serve to avoid any confusion between mnemiopsins and the *Mnemiopsis *opsin genes discussed in this paper. Furthermore, none of the 10 photoproteins were exact nucleotide matches to *mnemiopsin 1 *or *2 *from Jafarian *et al. *[21]. *Mnemiopsin 1 *is most similar *to MleiPP1 *(98.5% nucleotide identity (612 out of 621 bp)). *Mnemiopsin 2 *is most similar to *MleiPP5 *(97.3% nucleotide identity (607 out of 624 bp)). The 10 sequences have been deposited [GenBank:JQ724636-JQ724645]. They also have been assigned identifiers specific to the *Mnemiopsis *Genome Project (*MleiPP1-10*: ML085715b, ML085730b, ML085731a, ML085732b, ML085733a, ML085734b, ML085741b, ML215420b, ML215421a, ML215422b).

One cluster of photoprotein genes located on genomic scaffold ML0857 [GenBank:JH153500.1] includes seven full-length predicted genes, with six found in tandem in a head-to-tail orientation (Figure 1). These six genes are spaced at regular intervals (average 463.6 ±13.5 bp intergenic sequence). There are also two partial photoprotein gene predictions (incomplete gene fragments) on ML0857, each containing a gap in the scaffold where sequence is missing between two contigs joined in the draft assembly. These two partial predictions are located in tandem with a seventh full-length photoprotein gene on ML0857 (*MleiPP1*). A second cluster located on scaffold ML2154 [GenBank:JH154797.1] has three full-length photoprotein genes found in tandem, also with head-to-tail orientation and regular spacing (average 428 ± 8.5 bp intergenic sequence) (Figure 1). There is one partial predicted photoprotein gene on ML2154 that is located directly next to *MleiPP10*. A third scaffold, ML3841 [GenBank:JH156484.1], had one full-length photoprotein gene plus three partial predictions. A fourth scaffold, ML3423 [GenBank:JH156066.1], had one partial prediction. The predictions on ML3841 and ML3423 have very high sequence similarity to the genes on ML2154. The short length of these scaffolds (ML3841 is 4,191 bp; ML3423 is 1,704 bp) and the sequence similarity with genes on ML2154 strongly suggested that these sequences should be assembled with ML2154. When the individual sequence reads used to assemble ML3841 and ML3423 were analyzed manually using Consed (see Methods), we found multiple reads that had sequence exactly matching that on ML2154. We concluded that these scaffolds represent misassemblies by the Phusion assembler and that they should, in fact, be assembled with scaffold ML2154. Thus, we did not include sequences from ML3841 or ML3423 in any further analyses and have not included them in Figure 1.

### Confirmation of individual *Mnemiopsis *photoprotein sequences

Misassemblies of genomic scaffolds can result from the presence of multiple copies of closely related repetitive sequences that inappropriately collapse onto one or a few regions [47]. Alternatively, false gene duplications or expansions can also occur due to assembly errors [48]. Because of the complex nature of assembling genomic regions with a high level of repetitive sequence, as seen in the photoprotein gene clusters, we chose to confirm the presence of individual photoprotein genes using two complementary approaches: a 5' and 3'-rapid amplification of cDNA ends-PCR (RACE-PCR) screen; and a manual inspection of the individual sequence reads used to assemble each photoprotein gene in the genome. Alignments of the sequences obtained from the 5'-RACE-PCR screen (including UTR sequence) with the predicted photoprotein genomic sequence successfully confirmed seven out of ten of the predicted full-length photoprotein genes on ML0857 and ML2154 (Figure 1). The 3'-RACE-PCR screen confirmed one full-length photoprotein gene on ML2154 and one partial photoprotein gene on ML2154. Overall, the RACE-PCR screening approach confirmed eight of ten (80%) full-length photoprotein genes and one of three (33%) partial photoprotein genes predicted from the draft assembly. This gave us confidence that most of the identified photoprotein genes are not only present but are also transcribed in *Mnemiopsis*, indicating that they are likely functional genes. We suspect that the partial photoprotein genes are likely full-length genes, but that the full sequence cannot be determined from the current data. Further refinements of the draft assembly and targeted re-sequencing may help to determine the full-length sequence of these partial photoprotein genes. Another result obtained from both the RACE-PCR screen and the manual inspection was that two predicted full-length photoprotein genes (*MleiPP8 *and *MleiPP10*), which had single nucleotide deletions leading to frame shifts in the draft assembly, have been correctly annotated, confirming that they are full-length photoprotein genes rather than pseudogenes. Another predicted gene that contained a 7 bp sequence gap in the draft assembly (*MleiPP7*) was finished using data from both the RACE-PCR screen and the manual inspection, confirming another full-length photoprotein gene.

The manual inspection of the assembly confirmed that there was evidence (multiple high quality reads) supporting all 10 of the predicted photoprotein genes, therefore confirming 10 out of 10 full-length genes. Examining the individual reads also allowed us to identify polymorphic sites within each predicted protein that represent allelic variants of each (data not shown). In a few cases, examination of the flanking region surrounding photoprotein genes indicated high levels of variation beyond what would be expected for allelic variants, suggesting that there may be some photoprotein sequences that have been inappropriately collapsed into a single region. This is why we have chosen to report that there are 'at least' 10 full-length photoprotein genes in *Mnemiopsis*.

We hypothesize that clusters of photoprotein genes allow *Mnemiopsis *to quickly produce and maintain a large quantity of photoprotein (and thus light, as long as luciferin is not limiting) by producing transcripts at a faster rate. Observations of *Mnemiopsis *indicate that, unlike in most bioluminescent systems, it is difficult to exhaust the capacity of whole animals for light production by continuous stimulation [35]. In addition, it has been noted that, as the frequency of stimulation is increased, total light production by *Mnemiopsis *does not decrease [43]. These observations led Chang [43] to conclude that '[t]his probably indicates that photogenic material is continuously being produced.' This could easily be achieved by having multiple functional photoproteins encoded within the *Mnemiopsis *genome, as described above.

### Green fluorescent protein is not present in the *Mnemiopsis *genome

Calcium-activated photoproteins are often co-localized in photocytes with GFP family members, such as in *Aequorea *and *Obelia *[49], allowing the wavelength of bioluminescent light emission to be shifted from blue to green. Photocytes themselves can also be autofluorescent, partially due to blue fluorescence in the spent photoprotein [50,51]. The autofluorescence is different from the fluorescence of GFP, which has not been observed in *Mnemiopsis *[8]. We performed several BLAST searches with various GFP query sequences but did not find evidence for any GFP homologs in the *Mnemiopsis *genome. This is consistent with previous evidence that *Mnemiopsis *is not fluorescent and that its bioluminescence emission spectrum shows no sign of characteristic GFP-type emission.

### Sequence analysis of the *Mnemiopsis *photoproteins

Each of the 10 *Mnemiopsis *photoproteins are 206 or 207 amino acids in length. We generated a table of percentage amino acid sequence identity and similarity among full-length photoproteins and their close homologs (Additional file 1). Among *Mnemiopsis *photoproteins, there is 87% to 100% protein sequence identity. *Mnemiopsis *photoproteins share 85% to 91% sequence identity with other ctenophore photoproteins (*Beroe *and *Bolinopsis*). Ctenophore photoproteins share just 21% to 24% sequence identity and 41% to 46% sequence similarity with known hydromedusan cnidarian photoproteins. Within all hydromedusan photoproteins there is 60% to 94% sequence identity. We identified two photoprotein-like sequences in the cnidarian *Nematostella vectensis *(*NvecPP1*, GenBank:XM_001639610 and *NvecPP2*, GenBank:XM_001639611), which code for proteins that share 25% to 27% amino acid sequence identity with ctenophore photoproteins and 20% to 23% amino acid sequence identity with hydromedusan photoproteins. We identified four photoprotein-like sequences in the poriferan *Amphimedon queenslandica *(*AquePP1*-*AquePP4*; JGI ID:Aqu1.225927, Aqu1.223059, Aqu1.223058, Aqu1.222695), which code for proteins that share 15% to 25% amino acid sequence identity with ctenophore photoproteins and 16% to 27% amino acid sequence identity with hydromedusan photoproteins. The predicted molecular weight for the *Mnemiopsis *photoproteins ranges from 24.56 to 24.76 kDa and the predicted isoelectric point for this set of proteins ranges from pH 4.57 to 4.82 (Additional file 2).

**Figure 2:**
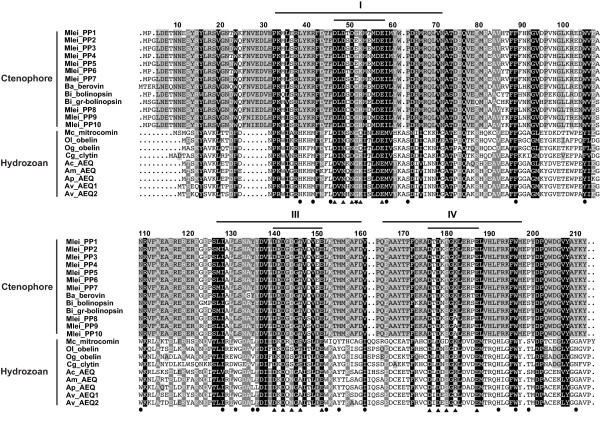
**Full-length amino acid alignment of functional photoproteins from hydromedusae and ctenophores**. EF-hand domains I, III, and IV are indicated by long lines above the alignment, while the 12-residue calcium binding loops within each EF-hand domain are indicated by short lines above the alignment. Calcium-coordinating residues within EF-hand domains (black triangles) and residues in the coelenterazine binding pocket (black circles) are indicated. Residue 6 within the EF-hand I calcium loop is also indicated (black star). Residue numbering is based on the berovin sequence (Ba_berovin). Columns of residues are shaded by similarity group conservation (defined by GeneDoc and the BLOSUM62 matrix) where black shows ≥90%, dark grey shows ≥70% and light grey shows ≥55% similar residues in a column. Species are abbreviated as follows: Mlei = *Mnemiopsis leidyi*; Ba = *Beroe abyssicola*; Bi = *Bolinopsis infundibulum*; Mc = *Mitrocoma cellularia*; Ol = *Obelia longissima*; Og = *O. geniculata*; Cg = *Clytia gregarium*; Ac = *Aequorea coerulescens*; Am = *A. macrodactyla*; Ap = *A. parva*; Av = *A. victoria*.

An alignment of photoproteins from hydromedusan cnidarians and ctenophores reveals several regions of sequence similarity (Figure 2). Specific residues that make up the coelenterazine binding cavity and the EF-hand domains (including the 12-residue calcium binding loops and the main loop ligand residues that coordinate calcium binding) are noted on the alignment. Within the MleiPPs, there is conservation of nearly all of these key functional residues, with a few notable exceptions. One interesting substitution in EF-hand I of all Sequence Group B sequences (noted by Jafarian *et al. *[21] for mnemiopsin 2) is a glutamic acid residue at residue 6 of the loop instead of the conserved glycine that is found in this position in each of the EF-hands of all other photoproteins. Also, compared with other *Mnemiopsis *photoproteins, Sequence Group C has a substitution in residue 7 of EF-hand IV (alanine instead of lysine), indicating that this calcium coordinating position may tolerate this substitution without significantly disrupting function. Other differences in key residues between *Mnemiopsis *sequence groups are either conservative substitutions that are likely to retain function or are also seen in hydromedusan photoproteins (for example, residue 7 of EF-hand I).

Comparing hydromedusae and ctenophore photoprotein protein sequences, similarity is especially high within the three EF-hand domains (Figure 2). At the N-terminus, there are two regions where the ctenophore photoproteins have insertions of six to nine amino acids relative to the hydromedusae photoproteins. There are several differences in important functional residues, especially in residues that make up the coelenterazine binding cavity, between the two groups of photoproteins, which have been previously discussed [20]. These amino acid substitutions may be partially responsible for the functional differences seen between the two groups, such as the property of photoinactivation present in ctenophore photoproteins but not in hydromedusae photoproteins. Future studies that examine functional and structural differences among photoproteins will help confirm what effect these and other residues have on function and determine what makes these two groups distinct.

### dN/dS analyses indicate strong purifying selection is acting on *Mnemiopsis *photoprotein genes

dN/dS analyses compare the rate of substitutions at synonymous (silent) sites (dS) to the rate of substitutions at nonsynonymous sites (dN) between a pair of coding sequences, and are used to examine rates of sequence evolution for evidence of natural selection at the molecular level [52]. If the dN/dS rate ratio is <1, there is evidence for purifying (negative) selection, and the duplicated genes are thought to be under selective constraint. Furthermore, the smaller the dN/dS ratio, the greater the selective constraint under which the two genes have evolved. The rate of nonsynonymous substitution is generally much lower than that of synonymous substitution because changes at coding sites are often constrained, as they may alter protein function [52]. We performed a maximum likelihood estimation of pairwise dN/dS ratios for all *Mnemiopsis *photoprotein gene pairs. For these genes, the ratios range from 0.0184 to 0.1691 (Table 1), indicating that strong purifying (negative) selection is acting on these sequences and that the function of the duplicated gene copies is being selectively maintained.

**Table 1:** Maximum likelihood estimation of pairwise dN/dS for the *Mnemiopsis *photoproteins.

Sequence Group Comparison	Seq1	Seq2	dN	dS	dN/dS
Within Group A	MleiPP1	MleiPP4	0.0192	0.1441	0.1333
	MleiPP1	MleiPP6	0.0058	0.2321	0.0252
	MleiPP4	MleiPP6	0.0953	0.0183	0.192
Within Group B	MleiPP2	MleiPP3	0.0138	0.2272	0.0607
	MleiPP2	MleiPP5	0.0038	0.2042	0.0184
	MleiPP3	MleiPP5	0.0181	0.3171	0.0572
	MleiPP2	MleiPP7	0.0293	0.2441	0.1201
	MleiPP3	MleiPP7	0.0188	0.111	0.1691
	MleiPP5	MleiPP7	0.0251	0.2409	0.1044
Between Groups A and B	MleiPP1	MleiPP2	0.0536	0.6694	0.08
	MleiPP1	MleiPP3	0.0386	0.493	0.0783
	MleiPP2	MleiPP4	0.0438	0.809	0.0541
	MleiPP3	MleiPP4	0.029	0.5159	0.0563
	MleiPP1	MleiPP5	0.0525	0.7689	0.0683
	MleiPP4	MleiPP5	0.0391	0.7813	0.0501
	MleiPP2	MleiPP6	0.0521	0.8626	0.0604
	MleiPP3	MleiPP6	0.0371	0.4546	0.0816
	MleiPP5	MleiPP6	0.0518	0.8676	0.0597
	MleiPP1	MleiPP7	0.0548	0.5394	0.1015
	MleiPP4	MleiPP7	0.0397	0.489	0.0813
	MleiPP6	MleiPP7	0.0532	0.5037	0.1057
Within Group C	MleiPP8	MleiPP9	0.0019	0.0837	0.0226
	MleiPP8	MleiPP10	0.002	0.0328	0.0623
	MleiPP9	MleiPP10	0.0039	0.0302	0.1287

### Evidence of concerted evolution within the *Mnemiopsis *photoprotein family

The evolution of multigene families can be explained by different models, including birth-and-death evolution and concerted evolution [53]. In birth-and-death evolution, new genes are created by gene duplication, with some duplicate genes remaining in the genome, whereas others are inactivated (converted to pseudogenes) or deleted from the genome outright. In concerted evolution, all family member genes evolve as a unit. A mutation occurring in a repeat spreads throughout family members via recombination events such as unequal crossover or gene conversion. The evolution of tandemly arrayed multigene families has often been attributed to concerted evolution. Current evidence supports this evolutionary model for some families, including many RNA genes in prokaryotes and eukaryotes, but in others, new analyses have shown that they are subject to birth-and-death evolution with strong purifying selection (for example, histone genes); still others are subject to a mixed process of concerted and birth-and-death evolution (for example, alpha-like globins) [53].

We tested the *Mnemiopsis *photoproteins for evidence of recombination events indicative of concerted evolution using GENECONV and found some evidence to support this model - namely, nine pairs of sequences on scaffold ML0857 with evidence for recombination from a global comparison of fragments (Additional file 3). The top three resulting pairs (*MleiPP3 *and *MleiPP6*; *MleiPP5 *and *MleiPP7*; and *MleiPP2 *and *MleiPP7*) are all within-sequence group pairs that have global permutation-test *P*-values and global Bonferroni-corrected Karlin-Altschul *P*-values <0.05. Our results suggest that the high level of sequence conservation in this multigene family is likely maintained through a combination of strong purifying selection and mechanisms of concerted evolution, though further analysis will be required to determine the relative contribution of each process.

### Analysis of photoprotein-like genes in the genomes of *Amphimedon *and *Nematostella*

We aligned the EF-hand domains coded by ctenophore, anthozoan cnidarian (*Nematostella)*, hydrozoan cnidarian, and poriferan (*Amphimedon) *photoprotein and photoprotein-like genes (Additional file 4). We analyzed the two *Nematostella *photoprotein-like sequences and found that some key residues have been substituted that likely disrupt calcium binding and, presumably, any ability to function as a luciferase. In the calcium-binding loop of EF-hand I, the first aspartic acid (loop position 1) has been replaced with a valine in both sequences. In EF-hand IV, the second aspartic acid (loop position 3) in the calcium-binding loop has been replaced with a lysine in both *Nematostella *sequences. These substitutions are not conservative and may be disruptive enough to prevent calcium from binding to these proteins. In fact, HMMER searches of the Pfam and SMART databases do not predict the presence of EF-hand IV in the *Nematostella *sequences, likely due to the disruptive substitution we identified in this domain.

We also analyzed the four *Amphimedon *(poriferan) sequences and found that none of the key residues in the calcium binding loops have major substitutions (Additional file 4). This suggests that these sponge proteins may have retained a calcium binding function, although the ability to produce visible light may not be intact given that there are no reports of light production from this species. In EF-hand I, a glutamic acid residue replaces the final aspartic acid (loop position 12) in three *Amphimedon *sequences. This is, however, a very conservative substitution, as both aspartic acid and glutamic acid have acidic side chains, and thus would probably not significantly disrupt calcium binding. HMMER searches of Pfam successfully identify EF-hands I, III, and IV in all four *Amphimedon *photoprotein-like sequences. Aque_PP2 has some minor differences compared with the other sponge proteins in key binding loop residues, but all of the residues that are substituted are found in at least one other functional photoprotein (Additional file 4).

Although we found that *Amphimedon *has four photoprotein gene homologs, visible bioluminescence has not been substantiated for that or any other sponge [1]. Ultra-weak luminescence has been reported for *Suberites domuncula *and that species also possesses an acyl-coenzyme A synthetase gene, which is similar to firefly luciferase [54]. However, this series of findings is not conclusive evidence of a functional bioluminescence system in Porifera, using either coelenterate- or firefly-type luciferin. Bioluminescence has also not been observed in the cnidarian *Nematostella*, yet we found that these organisms have photoprotein-like genes. There are at least three possible scenarios that might explain their existence in these organisms: these are functional photoprotein genes that are either rarely utilized or are used at stages or in situations that have not been witnessed by researchers; these genes encode calcium-binding proteins with functions not involved in bioluminescence; or they represent a kind of evolutionary intermediate or 'proto-photoprotein' that does not have all of the structural machinery in place to allow the protein to properly fold or coordinate calcium ion binding. In the case of the two *Nematostella *genes, there are substitutions in the EF-hand domains that make it unlikely that they can properly bind calcium. In the case of *Amphimedon*, it is possible that the genes encode functional photoproteins, but that these gene products are ultimately inactive because *Amphimedon *may not synthesize coelenterazine or obtain coelenterazine from its diet. This hypothesis could be tested by performing a luciferase activity assay on the purified proteins.

### *Nematostella *photoprotein-like mRNA expression

*In situ *hybridizations of two photoprotein-like genes from *Nematostella *during a series of developmental stages reveal a pattern of mainly endodermal expression, with a specific region of endodermal expression in the tips of budding tentacles through the six-tentacle stage (Additional file 5). The expression pattern of the two genes is not entirely overlapping. For example, *NvecPP2 *has a diffuse pattern of expression in the endoderm in the larval stage (Additional file 5, Panel A), whereas the corresponding pattern for *NvecPP1 *shows a distinct endodermal expression pattern in the developing tentacle buds (Additional file 5, Panel B). Additionally, for *NvecPP2 *only, at the six-tentacle stage, there is some expression in the basal disc of the anemone. Given that *Nematostella *does not exhibit bioluminescence, this pattern may indicate that these genes acquired a role in development. It would be informative to look at the expression pattern of the *Amphimedon *genes during various life stages as well, given that there is evidence in publicly available EST sequences (EST Database at the National Center for Biotechnology Information (NCBI)) that all four *Amphimedon *photoprotein-like genes are expressed during the larval stage. Expression patterns may help to narrow down possible functions for these proteins.

### Analysis of three putative *Mnemiopsis *opsins

We identified three putative *Mnemiopsis *opsin genes. These sequences were named *MleiOpsin1 *to *MleiOpsin3 *and have been deposited [GenBank:JQ724646-JQ724648]. They also have been given identifiers specific to the *Mnemiopsis *Genome Project (*MleiOpsin1 *to *3*: ML13055a, ML12047a, ML215412a). The protein encoded by *MleiOpsin1 *is 345 amino acids in length and the gene is comprised of eight exons. The protein encoded by *MleiOpsin2 *is 400 amino acids in length and the gene has 11 exons. The protein encoded by *MleiOpsin3 *is 404 amino acids in length and the gene has seven exons. Full-length cDNA sequences of the ORFs of *MleiOpsin1 *and *MleiOpsin2 *were extended by RACE-PCR. We generated a table of percentage amino acid sequence identity and similarity among the *Mnemiopsis *opsins and their close homologs based on the transmembrane region alignment (Additional file 6). The *Mnemiopsis *opsins share 17% to 36% amino acid identity and 38% to 59% amino acid similarity. MleiOpsin1 shares the highest percent identity with an opsin from *Pleurobrachia pileus*, another ctenophore species (PpilOpsin1, 37%, based on truncated sequence; see Additional file 6). MleiOpsin2 shares the highest percent identity with PpilOpsin2 (48%). MleiOpsin3 shares the highest percent identity with human peropsin (21%).

We analyzed MleiOpsins1 to 3 in relation to several residues known to be important to opsin function and found that MleiOpsin2 has retained all but two of these important amino acids, while MleiOpsin1 and 3 have additional substitutions (Additional file 7). Lys296 is a residue that serves as the site for the Schiff base linkage with the chromophore and is conserved in all known opsins, including MleiOpsins1 to 3. The counterion is a key functional residue within the opsin family, responsible for stabilizing the inactive dark state pigment by helping to stabilize the protonated Schiff base and tuning the wavelength absorbance into the visible spectrum [55]. The counterion is usually Glu113 (sometimes Asp113) in vertebrate visual and non-visual opsins and Glu181 in many other opsins. Interestingly, in MleiOpsins1 and 2, there is a Glu in both positions, suggesting that the ancestral metazoan opsin may have had a Glu at both positions. In MleiOpsin3, Glu is retained only in position 181, suggesting that, if functional, this protein may have the ability to stabilize the protonated Schiff base through this position. A disulfide bond, conserved in most G-protein coupled receptors, normally found at residues Cys110 and Cys187, is not present in MleiOpsins 1 to 3, presenting the possibility that either the proteins can fold and function properly without this conserved bond, or that their function is disrupted due to the substitution of these residues. Finally, conserved motif Glu134-Arg135-Tyr136, involved in the propagation of the transduction signal once a photon has been absorbed, is retained in MleiOpsin2, partially substituted to Glu-Gln-Tyr in MleiOpsin1 and fully substituted to Arg-Arg-Ala in MleiOpsin3. Overall, MleiOpsin2 has retained many conserved functional residues and has the greatest potential to be a functional opsin. This is further substantiated by the cloning and characterization of MleiOpsin2 (see the section on *Opsin protein expression and characterization*).

### Phototransduction pathway components are present in *Mnemiopsis*

Ciliary photoreceptors (typically associated with vertebrates) employ a phototransduction cascade that includes ciliary opsins, Gi/Gt proteins, phosphodiesterase, and cyclic nucleotide gated ion channels, whereas rhabdomeric photoreceptors (typically associated with invertebrates) utilize a cascade involving rhabdomeric opsins, G-protein alpha-q, phospholipase C, and transient receptor potential ion channels. Both cascades can be deactivated by arrestin and rhodopsin kinase, and regenerated by retinal binding protein. We searched for and identified putative homologs to several ciliary and rhabdomeric phototransduction pathway proteins [46] in the *Mnemiopsis *genome (Table 2). These sequences have been deposited into GenBank [GenBank:JQ724649-JQ724657, JX564543-JX564553]. The *Mnemiopsis *homologs had BLAST hits to query sequences with significant *E*-values (Table 2). Their reciprocal best BLAST hits were to proteins with annotations that closely correspond to the query proteins in nearly all cases. Exceptions include a guanine nucleotide-binding (G) protein specific to the ciliary pathway (G-alpha-t), which resulted in a top hit of G(i) subunit alpha-2; the guanylyl cyclase GC-E precursor, which gave a top hit of a natriuretic peptide receptor; and retinal-binding protein, which gave a top hit to a SEC14-like protein. Overall, these results indicate that components comprising a complete ciliary phototransduction cascade are present in *Mnemiopsis*. In addition, assembled RNA-seq transcript data available through the *Mnemiopsis *Genome Project Web site [56] via the 'CL' (Cufflinks) track on the *Mnemiopsis *genome browser support the developmental mRNA expression of all but two of the identified sequences (Table 2).

**Table 2:** *Mnemiopsis *homologs to ciliary and rhabodmeric phototransduction cascade components and their reciprocal best BLAST hit.

Protein name (GenBank accession number of query protein)	GenBank accession number of top *Mnemiopsis *result (ML identifier)	*E*-value	Reciprocal best BLAST query result Protein name [Species; GenBank accession number]	*E*-value	RNA-seq Evidence?
**Ciliary components**					

Opsin (ACB05673)	JQ724646 (ML13055)	2e-22	Opsin-3-like [*Oreochromis niloticus*; XP_003441288]	6e-31	Yes
G-alpha-s subunit (BAA81697)	JX564543 (ML012011)	1e-93	guanine nucleotide binding protein, alpha stimulating activity polypeptide [*Daphnia pulex*; EFX88427.1]	3e-177	Yes
G-alpha-i subunit (ACB05685.1)	JQ724654 (ML156514)	2e-129	G protein alpha subunit i class [*Halocynthia roretzi*; BAB79197.1]	2e-177	Yes
Transducin G-alpha-t1 (AAB01735_1)	JX564546 (MLRB156557)	9e-73	guanine nucleotide-binding protein G(i) subunit alpha-2 [*Mus musculus*; NP_032164.2]	3e-173	Yes
Transducin G-gamma-t1 (AAH25929_1)	JX564547 (ML17031)	7e-07	guanine nucleotide-binding protein G(T) subunit gamma-T1 [*Otolemur garnettii*; XP_003782721]	1e-06	Yes
GRK1 G protein-coupled receptor kinase 1 (AAH96611_1)	JX564550 (MLRB009169)	9e-110	G protein-coupled receptor kinase 5 [*Callithrix jacchus*; XP_002756686]	0.0	No
GMP-PDE alpha rod (NP_666198_1); GMP-PDE beta rod (P23440_3)	JX564548 (MLRB03248)	1e-97; 5e-99	sperm phosphodiesterase 5-like [*Saccoglossus kowalevskii*; XP_002733933]	0.0	Yes
GMP-PDE delta (O55057_1)	JX564549 (ML233327a)	6e-55	cGMP-specific rod phosphodiesterase 6D delta [*Trichoplax adhaerens; *XP_002113713]	8e-55	Yes
Phosphodiesterase (ACB05690)	JQ724657 (ML096829)	4e-105	High affinity cGMP-specific 3',5'-cyclic phosphodiesterase 9A-like [*Danio rerio*; XP_692819.2]	1e-163	Yes
Cyclic nucleotide gated ion channel (CAB42891.1)	JX564544 (ML08605) and JX564545 (ML054419)	2e-105 1e-115	cGMP-gated cation channel alpha-1-like, partial [*Cricetulus griseus*; XP_003515326] predicted protein [*Nematostella vectensis*; XP_001641603.1]	2e-124 1e-121	Yes
RGS9-1 regulator of G-protein signaling 9 isoform 1 (NP_035398_2)	JX564552 (MLRB369320)	2e-14	regulator of G-protein signaling loco-like [*Megachile rotundata*; XP_003705980]	1e-27	No
GC1 guanylyl cyclase GC-E precursor (NP_032218_2)	JX564553 (ML17474)	2e-131	natriuretic peptide receptor 1-like [*Saccoglossus kowalevskii; *XP_002734106]	0.0	Yes
Recoverin (NP_033064_1); GCAP1 guanylyl cyclase-activating protein 1 (NP_032215_2); GCAP2 guanylyl cyclase-activating protein 2 (NP_666191_1)	JX564551 (ML096819)	9e-51; 1e-32; 2e-38	Hypothetical protein DAPPUDRAFT_65663 [*Daphnia pulex*; EFX65172]	2e-121	Yes

**Rhabdomeric components**					

G-alpha-q subunit (ACB05683)	JQ724653 (ML009153)	4e-105	Heterotrimeric GTP-binding protein alpha subunit G-alpha-q [*Litopenaeus vannamei*; AAT44837.1]	8e-134	Yes
Phospholipase C (ACB05675)	JQ724649 (ML04921)	1e-178	Predicted protein [*Nematostella vectensis*; XP_001635876.1]	≤1e-1000	Yes
Trp-C protein (ACB05689)	JQ724656 (ML234550)	1e-11	Similar to TRP gamma cation channel [*Nasonia vitripennis*; XP_001604587.1]	1e-53	Yes

**Shared components**					

Visual G beta (ACB05681)	JQ724652 (ML02234)	7e-162	G-protein beta subunit [*Meloidogyne javanica*; ACB97665.1]	≤1e-1000	Yes
Rhodopsin kinase (ACB05677)	JQ724650 (ML04904)	8e-85	Beta-adrenergic receptor kinase 2 [*Danio rerio*; NP_001128197.1]	≤1e-1000	Yes
Arrestin (ACB05679 and P20443_1)	JQ724651 (ML047926)	4e-55	TRIADDRAFT_64255 [*Trichoplax adhaerens*; XP_002116188.1]	≤1e-1000	Yes
Retinal-binding protein (ACB05687)	JQ724655 (ML167044)	2e-28	Similar to SEC14-like protein 1 isoform 4 [*Canis familiaris*; XP_857362.1]	≤1e-1000	Yes

For each query protein, the corresponding *Mnemiopsis *protein model is listed with the *E*-value of the top BLAST result. The *Mnemiopsis *protein model was then used as a query in a reciprocal best BLAST search of the non-redundant protein database (NCBI) and the top result is listed along with the *E*-value of the BLAST result. RNA-seq data supports the developmental mRNA gene expression of each of these protein models. BLAST: Basic Local Alignment Search Tool; ML: *Mnemiopsis leidyi*; PDE: phosphodiesterase; TRP: transient receptor potential.

Ciliary phototransduction is more likely than rhabdomeric in *Mnemiopsis *because the putative photoreceptors in the apical sensory organ of ctenophores have a ciliary morphology [57], cyclic nucleotide gated ion channels (used in ciliary phototransduction) are the probable ancestral ion channels [58], and rhabdomeric phototransduction seems to have evolved with the emergence of bilaterians [58]. It would be interesting to determine the organismal function for the rhabdomeric phototransduction cascade components (G-protein alpha-q, phospholipase C and transient receptor potential ion channel genes) that we identified in the *Mnemiopsis *genome, and to determine if the rhabdomeric line of photoreceptor evolution also dates back to early metazoans. Although we have RNA-seq-based evidence that these genes are expressed during embryonic development, we do not know if their expression is limited to the photocytes and/or the four putative photoreceptors in the apical sense organ. Future studies that explore the expression patterns of these genes will help verify whether a phototransduction pathway is functioning in these specific regions and whether it is specific to ciliary components. Further analysis of the phototransduction pathway genes in this early branching metazoan will help shed light on the origin of these pathways and their function in a non-bilaterian animal.

### Metazoan phylogeny of photoproteins

**Figure 3:**
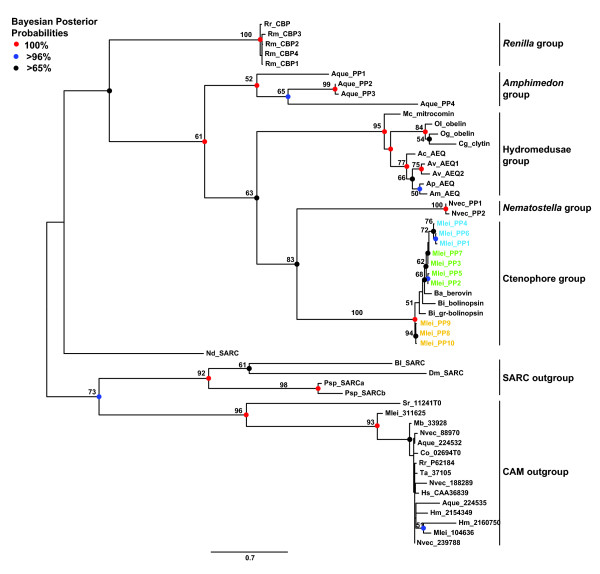
**Unrooted maximum likelihood phylogeny of photoprotein and photoprotein-like proteins showing clusters of major groupings**. Five major groups were reconstructed: ctenophoran, *Nematostella*, hydromedusan, and *Amphimedon *photoproteins, as well as closely related coelenterazine-binding proteins from *Renilla *(an anthozoan cnidarian). Sarcoplasmic calcium binding proteins (SARCs) and calmodulins (CAMs) from a variety of taxa branch outside of these five major groups. Bayesian methods reconstructed a tree with the same topology. Bootstrap support values greater than 50% are denoted. Bayesian posterior probabilities are shown as colored circles at nodes. Red circles indicate 100% support, blue circles indicate >96% support, and black circles indicate >65% support. Species are abbreviated as follows: Ac = *Aequorea coerulescens*; Am = *Amphimedon macrodactyla*; Ap = *A. parva*; Aque = *A. queenslandica*; Av = *A. victoria*; Ba = *Beroe abyssicola*; Bi = *Bolinopsis infundibulum*; Bl = *Branchiostoma lanceolatum*; Co = *Capsaspora owczarzaki*; Dm = *Drosophila melanogaster*; Hm = *Hydra magnipapillata*; Hs = *Homo sapiens*; Mb = *Monosiga brevicollis*; Mc = *Mitrocoma cellularia*; Mlei = *Mnemiopsis leidyi*; Nd = *Nereis diversicolor*; Nvec = *Nematostella vectensis*; Og = *Obelia geniculata*; Ol = *O. longissima*; Psp = *Penaeus *sp.; Rm = *Renilla mulleri*; Rr = *R. reniformis*; Sr = *Salpingoeca rosetta*; Ta = *Trichoplax adhaerens*.

The phylogeny based on full-length photoprotein amino acid sequence alignments yielded four major groups in a well-supported clade - specifically, ctenophoran, anthozoan cnidarian, hydrozoan cnidarian, and poriferan groups (Figure 3). Related proteins, including functionally related coelenterazine-binding proteins from *Renilla *(an anthozoan cnidarian), form a sister group to this clade. A sarcoplasmic calcium binding protein from the marine worm *Nereis diversicolor *(Nd_SARC in Figure 3) branches just outside the *Renilla *coelenterazine-binding proteins group. Other sarcoplasmic calcium binding proteins from a variety of taxa, as well as calmodulin proteins from various taxa (including metazoan outgroup taxa *Capsaspora, Monosiga*, and *Salpingoeca*, and non-bilaterian metazoan taxa *Amphimedon, Mnemiopsis, Nematostella, Hydra, Renilla*, and *Trichoplax*), form their own groups outside of this clade (Figure 3). The difference in likelihood values among resulting trees from the multiple runs was very small, indicating that our methods produced several nearly equally likely trees. The relationships presented in Figure 3 are maintained in the 50% majority rule consensus tree of all of the result trees (Additional file 8).

Interestingly, the cnidarian photoproteins are not monophyletic; the two *Nematostella *photoprotein-like genes branch next to the ctenophore photoproteins with moderately high support (83%), followed by the hydrozoan group and then by the four *Amphimedon *photoprotein-like genes, which fall at the base of all of the other photoprotein and photoprotein-like groups with low support (61%). It is not unusual for trees based on single genes to form clusters that are incongruous with taxonomy because of selection acting on a single locus, the presence of homoplasic characters, incomplete lineage sorting, or as a result of long-branch attraction [59,60].

Within the ctenophore photoproteins, the *Mnemiopsis *sequences do not form a monophyletic group (Figure 3). All sequences from scaffold ML0857 group together, with ML0857 Group A forming one subgroup (72% bootstrap) and ML0857 Group B branching in a ladder-like fashion from the A subgroup. The sequence from *Beroe *branches next to the *Mnemiopsis *ML0857 cluster with low support, followed by two sequences from *Bolinopsis*. The *Mnemiopsis *photoproteins on scaffold ML2154 form a subgroup (Group C) at the base of all of the other ctenophore photoproteins with 94% support. Bioluminescence is widespread among ctenophores [61], however, since genomic data are not yet available for any other ctenophore species, it remains to be seen if tandemly arrayed clusters of photoprotein genes will be identified in other ctenophores. The availability of genomes or transcriptomes from additional species will provide the basis for a more complete understanding of photoprotein evolution within the Ctenophora. We also note that the ctenophore photoproteins are quite distinct from the hydromedusae photoproteins. Our phylogenetic tree separates these two groups into distinct clusters; however, the groups are clearly evolutionarily related compared with other calcium-binding proteins, indicating that there may have been relatively rapid diversification of these proteins in the time since ctenophores and cnidarians diverged (Figure 3).

### Photoproteins arose at the base of the Metazoa

**Figure 4:**
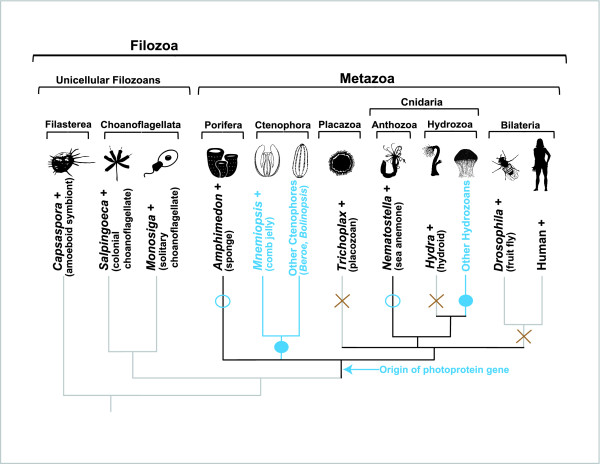
**Evolutionary history of the calcium-regulated photoprotein family**. According to currently available sequence data, the family arose at the base of the Metazoa. Filled blue circles indicate that the genes are present and that bioluminescence is observed in these groups. Open blue circles indicate that the genes are present, but that bioluminescence is not observed in these groups. A brown X indicates that photoprotein genes are absent from those groups. A plus sign next to a taxon name indicates that this organism's genome has been fully sequenced.

Searches of the recently sequenced genomes of unicellular eukaryotes (filozoans), including *Monosiga brevicollis *and *Salpingoeca rosetta *(choanoflagellates), and *Capsaspora owczarzaki *(a filasterean), did not reveal any photoprotein genes. However, BLAST searches revealed that homologs to other EF-hand superfamily proteins are present in the genomes of these groups, including calmodulin (shown in Figure 3), centrin or caltractin, myosin, calcineurin, spectrin, and actinin (data not shown). We found that photoproteins are altogether absent from the placozoan *Trichoplax*. Photoproteins are known to be present in many hydrozoan cnidarians in the Orders Leptothecatae, Trachymedusae, Narcomedusae, and Siphonophorae, but it appears that they have been lost from another hydrozoan, *Hydra *(Order Anthoathecatae). Because photoproteins or photoprotein-like genes are present in the earliest branching groups (that is, sponges and ctenophores) on the animal tree of life, and not in their closest non-metazoan relatives, we infer that the origin of the gene family was at the base of the Metazoa, followed by lineage-specific losses in *Trichoplax *and *Hydra *(Figure 4). This conclusion is based on currently available sequence data and should be revisited when sequence data become available for additional bioluminescent non-metazoan eukaryotes, such as radiolarians, which may have homologous calcium-activated photoproteins [1].

### Opsin phylogeny reconstructs a ctenophore-specific clade

**Figure 5:**
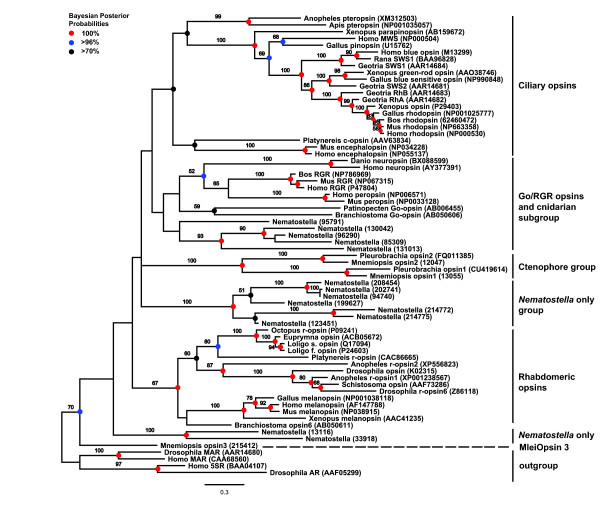
**Maximum likelihood phylogeny of opsin proteins showing clusters of major groupings**. Bayesian methods reconstructed a tree with the same topology. Bootstrap support values greater than 50% are denoted. Bayesian posterior probabilities are shown as colored circles at nodes. Red circles indicate 100% support, blue circles indicate >96% support, and black circles indicate >70% support. Gene name abbreviations: 5SR = fifth somatostatin receptor; AR = allatostatin receptor; MAR = muscarinic acetylcholine receptor; MWS = medium-wavelength sensitive; RGR = retinal G protein-coupled receptor; Rh = rhodopsin; SWS = short-wavelength sensitive.

We identified six major groupings in the opsin phylogeny we reconstructed (Figure 5), which was based on an alignment of the seven-transmembrane (7TM) region of opsin sequences from a variety of taxa (alignment with a subset of taxa shown in Additional file 7). Two putative *Mnemiopsis *opsin sequences (MleiOpsin1 and MleiOpsin2) form a well-supported clade (99% bootstrap) with opsins from the ctenophore *Pleurobrachia*, falling outside of the well-defined ciliary, rhabdomeric, and Go-coupled plus retinochrome, retinal G protein-coupled receptor (Go/RGR) groupings of bilaterians. The ctenophore-specific clade branches closer to the ciliary-opsin and Go/RGR groups than to the rhabdomeric-opsins. A third putative *Mnemiopsis *opsin (MleiOpsin3) does not group with the other ctenophore opsins, and instead branches on its own at the base of all opsins with low support. The leaf stability index (0.421) and branch attachment frequency value (0.30) are both very low for this branch, further supporting the uncertainty of the placement of this sequence in our phylogeny. Cnidarian opsins, represented by 14 *Nematostella *sequences, are found in three major groupings: a subgroup that falls next to the Go/RGR group; a group with seven *Nematostella *sequences that branches next to the ctenophore-specific clade; and a group with two *Nematostella *sequences that branches outside all opsins other than MleiOpsin3. Likelihood values among resulting trees from the multiple runs were all very similar, indicating that our methods produced several equally likely trees. The relationships presented in Figure 5 are maintained in the 50% majority rule consensus tree of all of the result trees (Additional file 9). We also repeated the analysis after removing MleiOpsin3 and all of the groupings and relationships among the groups remained the same (Additional file 10).

The major clades identified in our opsin tree are in agreement with results seen in recent studies [62-64] where cnidarian opsins occur in multiple locations across the tree. One recent study by Porter and colleagues did not obtain this result, instead finding that all cnidarian and one ctenophore opsin (PpilOpsin1) form a monophyletic clade referred to as Cnidops [65]. In that study, only three *Nematostella *sequences (all from the same subclade, with no representatives from the other two previously reported subclades) and a single ctenophore sequence were included in the analysis; by contrast, 14 *Nematostella *and four ctenophore sequences were included in our own analysis. The Porter study also included several putative opsin sequences from the cnidarian *Hydra *that we did not include in our analysis due to the extraordinarily long branches produced by these sequences during our preliminary phylogenetic analyses. Additionally, the single ctenophore sequence included in that study (PpilOpsin1) was derived from EST data and is truncated. These differences could explain why the branching pattern seen in Porter *et al. *[65] differs from the position seen in our own tree. Overall, the phylogeny we reconstructed does not suggest a simple one-to-one correlation between the non-bilaterian (ctenophore and cnidarian) opsin groups and the well-defined bilaterian groups. With the addition of the ctenophore sequences to the opsin phylogeny, the evolutionary path leading from prebilaterian opsins to the bilaterian ciliary, rhabdomeric, and Go/RGR opsins remains unresolved, and it appears that additional sequence data will be required to fully resolve these relationships.

### *Mnemiopsis *photoprotein expression and spectral analysis

**Figure 6:**
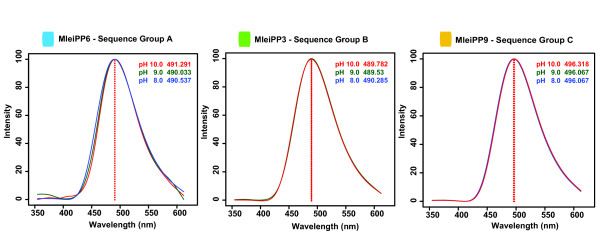
**Spectrum of bioluminescent emission for a subset of *Mnemiopsis *photoproteins representing the three sequence groups (A, B, C) at pH 8.0, 9.0, and 10.0**. The color of each curve corresponds to the key shown in the upper right of each graph.

We expressed, purified, and characterized a subset of the *Mnemiopsis *photoproteins that included one protein from each sequence group: MleiPP6 (Group A), MleiPP3 (Group B), and MleiPP9 (Group C). The emission wavelength maximum (λ_max_) at pH 8.0 for MleiPP6 and MleiPP3 was 490 nm and for MleiPP9 was 496 nm. All emission wavelengths were maintained within 1 nm at pH 8.0, 9.0, and 10.0 (Figure 6) for each protein. Although noteworthy, the slightly longer emission maximum for the Group C photoproteins would likely not have a functional impact on any organism detecting the luminescence. These values are slightly shifted from the *in vitro *value of mnemiopsin-1 and mnemiopsin-2 presented by Ward and Seliger [28] of 485 nm, which were determined at pH 8.4 and pH 8.3 respectively. This could be due to differences in pH and the purification methods used between the two studies. Although we only expressed three of the MleiPPs, it is likely that all 10 are functional, considering the very high amino acid identity present within each sequence group and, in particular, the conservation of key functional residues across all *Mnemiopsis *photoproteins. Although Group B sequences have a substitution in an important residue in EF-hand I, we show that one member of this group, MleiPP3, is a functional photoprotein, so it is likely that this substitution does not disrupt function in the other Group B proteins.

### *Mnemiopsis *opsin protein expression and characterization

**Figure 7:**
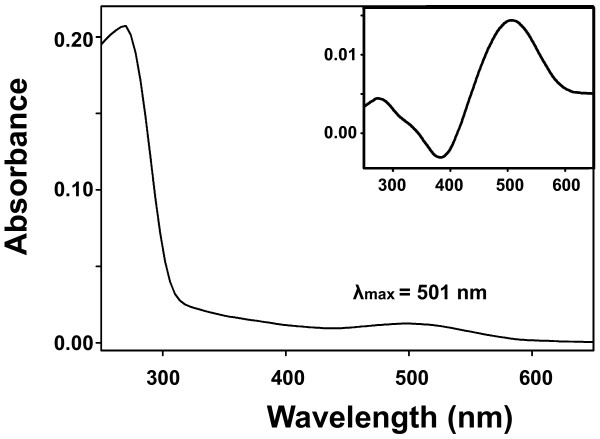
**Absorbance spectra of MleiOpsin2 pigment measured in the pre- and post-bleaching difference (inset) conditions**. In the latter case, the post-bleaching (light) spectrum was subtracted from the pre-bleaching (dark) spectrum.

We purified and determined the absorption spectrum of MleiOpsin2 bound to 11-*cis*-retinal. The wavelengths of maximal absorption (λ_max_) of the resulting visual pigment were measured from the dark spectrum and dark-light difference spectrum (Figure 7). This protein has an absorption peak at 501 ± 1 nm (Figure 7). When the regenerated pigment was exposed to light, it showed a new absorbance spectrum peak at around 380 nm (data not shown), indicating that 11-*cis*-retinal in the pigment was isomerized by light and all-*trans*-retinal was released. These results indicate that MleiOpsin2 is able to form a functional photopigment. The absorption spectrum overlaps with the emission spectrum of the photoproteins (Figure 6), suggesting that the opsin could function to absorb light produced by the ctenophore. Further experimentation will be necessary to rigorously test this hypothesis.

### *Mnemiopsis *photoprotein mRNA expression

**Figure 8:**
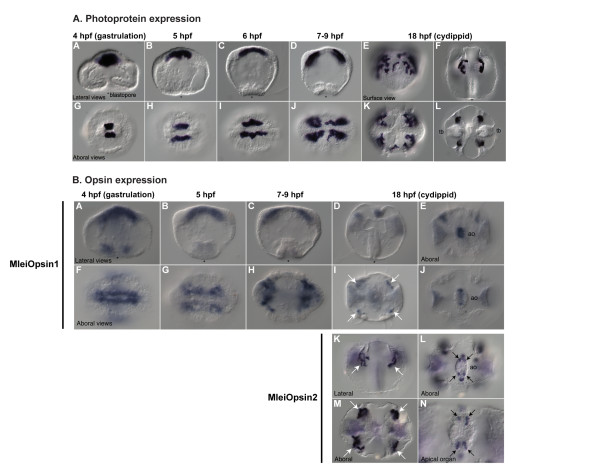
***In situ *hybridization showing *Mnemiopsis *photoprotein mRNA expression, and *Mnemiopsis *opsin mRNA expression**. **(A) **Photoprotein expression: Panels A-D and F are lateral views; E is a surface view; and G-L are aboral views. The blastopore is indicated with an asterisk. Tb = tentacle bulb. **(B) **Opsin expression: Panels A-D and K are lateral views; E, F-J, and L-N are aboral views. The blastopore is indicated with an asterisk. Ao = apical organ. White arrows indicate expression in regions of putative photocytes. Black arrows indicate expression in four putative photoreceptors in apical organ. hpf: hours post-fertilization.

We examined mRNA expression patterns of *Mnemiopsis *photoprotein genes through embryonic development by *in situ *hybridization. We used probes generated from each of the photoprotein sequence groups (A, B, and C); because the patterns were identical for all three probes, representative patterns are shown in Figure 8. Photoprotein expression begins during late gastrulation (4 to 6 hpf) in migrating photocyte precursors (Figure 8A, panels A-D, G-J) and continues in cells associated with the endodermal meridional canals beneath the developing comb plates where photocytes are located (Figure 8A, panels E-F, K-L). Expression coincides closely with the onset of light emission in developing photocytes, which starts around 8 hpf.

Although three separate probes were designed from the photoprotein sequences (one for each sequence group), identical expression patterns were observed from each probe. This could be due to true co-expression or may have resulted from the probes cross-hybridizing with multiple transcripts, given that the sequence is so similar among the photoproteins. The pattern of expression of *Mnemiopsis *photoproteins matches the site of light production in developing embryos, which begin to emit light in these same regions upon stimulation starting around 8 hpf [38], although mRNA expression is first detected at 4 hpf.

### *Mnemiopsis *opsin mRNA expression

Expression of two opsin homologs (*MleiOpsin1 *and *MleiOpsin2*) was detected in migrating photocyte precursors (*MleiOpsin1*) and in developing photocytes at 18 hpf (*MleiOpsin2*). *MleiOpsin1 *exhibits expression early in development that looks strikingly similar to the photoprotein expression pattern (Figure 8B, panels A-C, F-H), although it is not as strong or well-defined as the photoprotein expression pattern itself. Expression of *MleiOpsin1 *continues into the cydippid stage, where it is weakly expressed in photocytes (Figure 8B, panels D and I). *MlOpsin2 *is not expressed in early developmental stages (data not shown), but exhibits strong expression in photocytes that overlaps with photoprotein expression at the cydippid stage (Figure 8B, panels K and M, white arrows). At about 18 hpf, *MleiOpsin2 *expression is also found in four small groups of neural cells in the floor of the apical organ (Figure 8B, panels L and N, black arrows). These groups of neural cells coincide with structures described as lamellate bodies that were suggested over 130 years ago to be photoreceptors [56,66]. *MleiOpsin1 *is expressed in the apical sense organ at the cydippid stage as well, although it is not as well defined (Figure 8B, panels E and J). We did not examine expression patterns of *MleiOpsin3*. Overall, these patterns suggest that there are photoreceptors located in the apical sense organ and that photocytes may also function as photoreceptors in this species.

### *Mnemiopsis *photoprotein and opsin mRNA co-expression in photocytes

**Figure 9:**
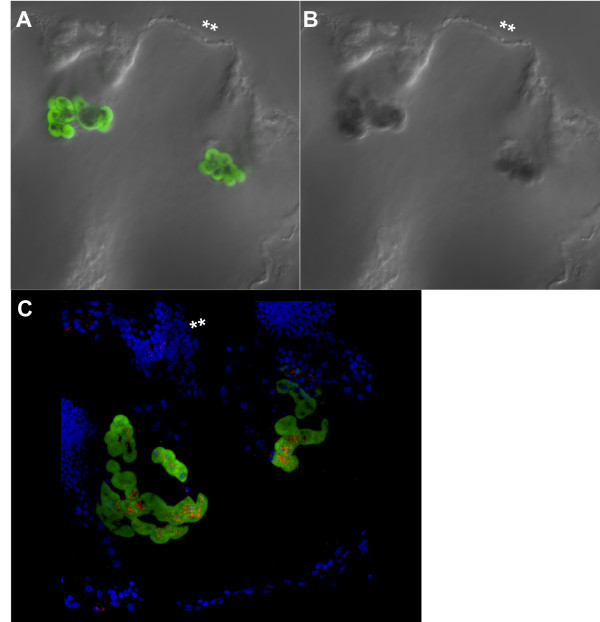
**Co-expression of *Mnemiopsis *photoprotein (*MleiPP1*) and opsin (*MleiOpsin2*) mRNA expression**. All images are lateral views of a cydippid approximately 18 hpf and a double asterisk denotes the aboral pole of the embryo. **(A) **Green staining is *MleiPP1 *mRNA expression via fluorescence *in situ *hybridization (fluorescein probe). **(B) **Dark blue staining is *MleiOpsin2 *mRNA expression via nitro blue tetrazolium (NBT) and 5-bromo-4-chloro-3-indolyl phosphate (BCIP) (digoxigenin probe). **(C) **Reflective confocal microscopy of co-fluorescent *in situ *embryo shown in (A) and (B). Bright blue staining is nuclei via 4'-6-diamidino-2-phenylindole; green staining is *MleiPP1 *mRNA expression via fluorescence *in situ *hybridization; red staining is *MleiOpsin2 *mRNA expression via NBT/BCIP.

Double *in situ *hybridization of photoprotein *MleiPP1 *and *MleiOpsin2 *shows a clearly overlapping expression pattern (Figure 9A,B). Reflective confocal microscopy of the same double *in situ *specimen (18 hpf) demonstrates that the *MleiOpsin2 *expression is punctate and is located within the same photocyte cells exhibiting photoprotein expression (Figure 9C). This pattern lends support for the hypothesis that photocytes function in bioluminescence and photoreception.

### Light responses in *Mnemiopsis*

Previous research has examined responses to light in *Mnemiopsis*, and there is some experimental evidence that light influences its spawning behavior. Under natural conditions, spawning normally occurs approximately 8 h after sunset in specimens collected in Woods Hole, MA, USA [67] and 3 to 5 h after onset of darkness in specimens collected in Miami, FL, USA [68,69], with the Miami specimens representing a genetically distinct population [70]. Thus, the signal to spawn corresponds to the absence of light or onset of darkness and may be temperature dependent. It is unclear, however, if *Mnemiopsis *exhibits phototactic behavior at any life stage ([71], KP, personal observation). The organismal function(s) of opsin-mediated phototransduction likely to occur in the photoreceptors of the apical sense organ and in the photocytes remains to be determined through experimentation.

### Do ctenophore photocytes have a dual role in light production and light sensing?

The co-expression of opsin and photoprotein genes in photocytes of *Mnemiopsis *suggests a dual role for photocytes in both light sensing and light production. Whether the two phenomena are functionally connected in this organism remains to be definitively determined through experimentation, although overlapping emission and absorption spectra of the two proteins suggests that it is a viable hypothesis. Although light-induced bioluminescence has been demonstrated previously in a few bilaterian taxa, including pyrosomes (Phylum Tunicata [72]) and in several crustaceans [73-76], the mechanism for this type of stimulation of light production has not been well investigated in any system. Further investigation will be required to determine whether light sensing stimulates light production in *Mnemiopsis *or if the two phenomena are linked in any way.

It is clear that *Mnemiopsis *bioluminescence is under neural control [36,37,39,43,44], but the co-localization of photoproteins and opsins in the same cells suggests that the cascade of events leading to and/or inhibiting bioluminescence may be more complex. Photoinactivation of photoproteins that is typical of ctenophores adds an additional layer of complexity to the system.

Here, we have shown that a ctenophore expresses a protein that senses the environment (light-sensing via opsin), and proteins that produce light (photoproteins), establishing that ctenophore photocytes are capable of sensing as well as responding to stimuli. Our results suggest that there is a non-visual function for opsin-mediated phototransduction in this early-branching metazoan species. Further study will be necessary to determine how the bioluminescence cascade operates in *Mnemiopsis*, and whether or not opsin and other phototransduction pathway genes play a role in regulating (promoting or inhibiting) luminescence production under different conditions.

## Conclusions

Here, we present a comprehensive analysis of the genes involved in light emission and photoreception in the ctenophore *Mnemiopsis leidyi*. With respect to light emission, we identified two genomic clusters of tandemly arrayed photoproteins genes in *Mnemiopsis *containing a total of at least 10 full-length genes with high sequence conservation; we have also shown that they are likely maintained through purifying selection and concerted evolution. We were able to confirm that the multiple photoprotein isoforms previously reported by others are, indeed, products of different (or separate) individual genes. Further, our understanding of the evolution of the calcium-regulated photoprotein gene family has significantly improved as a result of having full-length genomic sequence data from early-branching non-bilaterian taxa in-hand. Based on these data, we have constructed the first metazoan-wide phylogeny for the photoprotein gene family, identified photoprotein-like genes in non-luminescent taxa (the poriferan *Amphimedon *and the cnidarian *Nematostella*), and demonstrated that the family likely arose at the base of the Metazoa. Regarding photoreception, we identified three putative opsin genes in *Mnemiopsis*, reconstructed a phylogeny that suggests ctenophore opsins do not group clearly with traditional bilaterian rhabdomeric, ciliary, or Go/RGR opsin groupings, and have identified a complete ciliary phototransduction cascade in the *Mnemiopsis *genome. We have demonstrated that one ctenophore opsin (MleiOpsin2) forms a functional photopigment that absorbs light at wavelengths that closely overlap with maximum photoprotein light emission. We believe that MleiOpsin2 represents the most ancient animal opsin with a demonstrated ability to absorb light. Expression patterns showing co-localization of photoprotein genes and two putative opsin genes in developing *Mnemiopsis *photocytes indicate that these cells have the capacity to both sense and produce light. Opsin expression was also detected in the apical sensory organ in neural ciliary cells identified over 130 years ago as putative photoreceptors. This study sets the stage for future experimentation that will be necessary to test the intriguing possibility of a functional linkage between light reception and light production in this ctenophore species.

## Methods

### Identification of photoproteins in the *Mnemiopsis *genome

We used the *Mnemiopsis *draft genome, which was previously sequenced using 454 and Ilumina sequencing and assembled into scaffolds [32] using the Phusion assembler [77]. The current draft assembly comprises 5,100 scaffolds (scaffold N-50 of 123 kb), which corresponds to coverage of approximately 12X. A total of 16,645 protein-coding gene models were predicted by using a combination of FGENESH [78], PASA [79], and EVidenceModeler software [80], which combines *ab initio *gene predictions and protein and transcript alignments into weighted consensus gene structures. These data are publicly available at the *Mnemiopsis *Genome Project website [56].

Hydromedusan photoprotein homologs were used in TBLASTN and BLASTP searches of the *Mnemiopsis *genome assembly and predicted gene models, respectively. Candidate matches were then used as queries in a reciprocal BLASTP search against the non-redundant protein database (GenBank). Scaffolds containing putative photoprotein gene models were visualized in the JBrowse web-based genome browser [81,82].

### Confirmation of individual *Mnemiopsis *photoprotein sequences

Because several *Mnemiopsis *photoprotein gene predictions occur in tandem on the same scaffold, we sought additional evidence to determine how many full-length photoprotein genes are truly present and, of these, how many transcripts are actively expressed. We used two complementary methods to confirm the presence of a full-length *Mnemiopsis *photoprotein gene. First, we used a 5'- and 3'-RACE-PCR screening approach to determine which photoprotein transcripts are expressed during *Mnemiopsis *development. For this, we designed multiple gene-specific 5'- and 3'-RACE-PCR primers to target universally conserved regions of the coding sequence found among all the photoprotein gene models predicted from the assembly (Additional file 11). These primers were used in 5'- and 3'-RACE-PCR reactions (SMART RACE kit, Clontech Laboratories, Inc., Mountain View, CA, USA) with RNA from mixed developmental stages ranging from 0 to 30 hpf resulting from the spawn of several individuals. In some cases, one round of RACE-PCR was performed; in others, a second round of RACE-PCR with a second nested gene-specific primer was used. Individual RACE-PCR products were cloned and sequenced, and sequences were aligned to the genomic sequences using MacClade v4.08 [83]. Although the coding regions of all predicted *Mnemiopsis *photoproteins have high sequence identity, the sequence in the 5' and 3' UTRs differs substantially, which allowed us to positively identify which genomic sequence was identified by each product sequenced from the RACE-PCR screen. We considered aligned matches from either a 5'- or 3'-RACE sequence as confirmation that a particular transcript was expressed.

We obtained the second piece of evidence for the presence of putative photoproteins by examining the individual sequence reads that made up the Phusion assembly using Consed, which is a tool for viewing, editing, and finishing sequence assemblies [84,85]. Illumina and 454 sequence reads were mapped to Phusion scaffolds containing putative photoproteins using cross_match (P. Green, unpublished; [85]). The mapping results were used to generate an alignment file that could be utilized by Consed. The Consed assembly comprises a subset of 454/Roche fragment and Illumina mate-pair reads that map to the photoprotein gene family. The initial inspection of this assembly assessed general coverage and mate-pair consistency across the targeted regions. Any mate-pair inconsistencies and sequence gaps were noted. RACE-PCR sequences used to confirm the presence of individual *Mnemiopsis *photoproteins were then imported into the Consed assembly. The incorporated RACE sequences were then aligned to the assembled reads for comparison.

## Sequence analysis

### Photoproteins and opsins

Putative *Mnemiopsis *photoproteins were aligned to known photoprotein homologs using MUSCLE [86] and corrected manually to align specific residues within EF-hand domains. Putative opsin sequences were aligned to known opsins with MUSCLE and manually trimmed to the transmembrane region (approximately 270 amino acids). Alignments were highlighted by similarity group conservation (defined by GeneDoc and the BLOSUM62 matrix). For both the photoprotein (full-length alignment, Additional file 12) and the opsin alignment (7TM region alignment, Additional file 13), amino acid identity and similarity pairwise comparisons were calculated in BioEdit v7.0.5 [87] based on the alignments obtained with MUSCLE. Percentage identity was calculated as the percentage fraction identical characters in pairwise aligned sequences, treating gaps as an additional character. For similarity comparisons, the BLOSUM62 matrix was used. These alignments were used for phylogenetic analyses. Predicted molecular weights for the photoproteins were calculated using the Compute pI/Mw tool on the ExPASy Proteomics Server [88].

### dN/dS analyses to test for selection among *Mnemiopsis *photoprotein genes

We used the codeml program within the PAML software package [89] to generate maximum likelihood estimates of pairwise dN/dS (nonsynonymous/synonymous rate ratio, or omega) for the *Mnemiopsis *photoprotein genes. After testing various models, we selected the F61 model (CodonFreq = 3), which generates an empirical estimate of each codon frequency. We used a hill-climbing algorithm to maximize log-likelihood function with respect to kappa (transition/transversion ratio, fix_kappa = 0), t (sequence distance), and omega (fix_omega = 0). Using this approach accounts for transition/transversion rate biases, codon usage bias, and multiple substitutions. Analyses were conducted across the entire gene for all pairwise comparisons among the 10 sequences. All positions containing alignment gaps were eliminated automatically. Pairs of sequences with dS values (the number of synonymous substitutions per synonymous site) greater than 1.0 were considered too different from one another and discarded to avoid problems of mutational saturation [52]. Mutational saturation in DNA and protein sequences occurs when individual sites have undergone multiple mutations over time, causing the number of observed differences to no longer accurately reflect the true evolutionary distance, or number of substitutions that have actually occurred since the divergence of the two sequences [90]. This eliminated all pairwise comparisons between Sequence Groups A and C, and B and C.

### Testing the *Mnemiopsis *photoproteins for evidence of concerted evolution

We used GENECONV v1.81 [91] to test the *Mnemiopsis *photoproteins for evidence of recombination events that are indicative of concerted evolution. We changed the default gscale from 0 to 1, ignored all sites with missing data, and did a global analysis with 10,000 permutations and a *P*-value cutoff of 0.05.

### Searching for phototransduction pathway genes in *Mnemiopsis*

Phototransduction genes [46] were used as queries in BLASTP searches against the set of *Mnemiopsis *predicted gene models (version 2.2) as well as an unfiltered set of *Mnemiopsis *gene models (that is, gene predictions not incorporated into the final set). The identifier and the *E*-value of the top hit are listed in Table 2. A reciprocal best BLASTP search was performed using the top *Mnemiopsis *gene model against the non-redundant protein database in NCBI. The top result and the *E*-value of the hit are also listed in Table 2.

## Phylogenetic analysis

### Sequence retrieval for photoprotein phylogeny

Hydromedusae photoprotein homologs were used in TBLASTN and BLASTP searches of available genome assemblies and predicted gene models of non-metazoan eukaryotic phyla, including the choanoflagellates *Monosiga brevicollis *and *Salpingoeca rosetta*, the amoeboid symbiont *Capsaspora owczarzaki*, and non-bilaterian metazoan taxa including the poriferan *Amphimedon queenslandica*, the placozoan *Trichoplax adhearans*, and the cnidarians *Nematostella vectensis *and *Hydra magnipapillata*. For calmodulin outgroup sequences, human calmodulin [GenBank:CAA36839] was used in similar BLAST searches. An identical search strategy was used to query the *Mnemiopsis *genome and predicted gene models for calmodulin homologs, which resulted in two hits (Mlei_311625 and Mlei_104636 in Figure 3), which have been deposited [GenBank:JQ724658 and JQ724659]. SARC outgroup sequences were obtained from GenBank. The filtered protein models for *Monosiga *v. 1.0 [92], *Trichoplax *v. 1.0 [93], *Nematostella *v. 1.0 [94], and *Hydra *v. 1.0 [95] were downloaded from each species' Joint Genome Institute genome website. The set of *Amphimedon *gene models was downloaded from the ftp site provided in the genome paper [96]. Gene models for *Capsaspora *and *Salpingoeca *were downloaded from the Origins of Multicellularity Sequencing Project at the Broad Institute [97] in March 2011.

### Sequence retrieval for opsin phylogeny

A number of recent studies have described the diversity and phylogenetic relationships of opsins from various animals including cnidarians, protostomes, and deuterostomes [62-64,98]. A majority of these studies reconstructed phylogenies based on the functionally conserved 7TM region of the proteins. A subset of sequences reported in these studies was selected to represent the diversity of previously studied animal opsins, which include rhabdomeric, ciliary, and Go/RGR opsins, as well as including 14 *Nematostella *opsins to represent cnidarian-specific clades. Opsins from *Nematostella *and human were used in TBLASTN and BLASTP searches of genome assemblies and predicted gene models for *Trichoplax, Amphimedon, Monosiga*, and *Capsaspora*, as described above. Searches in genomes from these later four species yielded no positive hits to opsins, consistent with earlier analyses [62,64]. An identical search strategy was used to query the *Mnemiopsis *genome and predicted gene models, which resulted in three hits (*MleiOpsin1 *to *3*). We also searched available EST data at NCBI for *Pleurobrachia *sequences, which resulted in identifying one sequence (*PpilOpsin1*, GenBank:CU419614), which was truncated, and a second sequence (*PpilOpsin2*, GenBank:FQ011385), which covered the complete 7TM domain.

### Photoprotein and opsin phylogenies

To choose the best-fit model of protein evolution, we used the program ProtTest v2.4 [99] to apply Akaike information criterion 1 and 2 and Bayesian information criterion 2 metrics to a variety of possible substitution matrices and rate assumptions [100]. The results from the overall comparison of these metrics indicated the best fit model for the full-length photoprotein alignment was LG+I+Γ+F and for the opsin alignment was LG+Γ+F, where 'LG' indicates the substitution matrix [101], 'I' specifies a proportion of invariant sites, 'Γ' specifies gamma-distributed rates across sites, and 'F' specifies that empirical amino acid frequencies in the dataset are used.

Maximum likelihood analyses were performed with the MPI version of RAxML v7.2.8 (RAXML-HPC-MPI) [102]. For each alignment, we conducted two independent maximum likelihood searches: one with 25 parsimony starting trees (raxmlHPC-MPI -f d -m PROTGAMMAILGF -s input.phy -#25 -k) and another with 25 random starting trees (raxmlHPC-MPI -f d -m PROTGAMMAILGF -s input.phy -#25 -d -k). For all analyses, 100 bootstrapped trees were computed. Bayesian analyses were performed with MrBayes3.2 [103]. MrBayes does not support the LG model of evolution, so we used the second best fit model in ProtTest for each analysis (photoprotein: RtREV+I+ Γ +F; opsin: WAG+ Γ +F) with two independent five million generation runs of five chains, with trees sampled every 500 generations using the following execution block (prset aamodelpr = fixed(rtrev); lset rates = Invgamma; prset statefreqpr = fixed(empirical); mcmp mcmcdiagn = no nruns = 1 ngen = 5000000 printfreq = 5000 samplefreq = 500 nchains = 5 savebrlens = yes; mcmc;). Convergence diagnostics, examined with the help of AWTY [104], indicated a conservative burn-in fraction of 0.25. The runs all reached stationarity, and adjusting the burn-in did not affect the topology, swap rate, or other indices of convergence. Consensus trees and posterior probabilities were calculated once the stationary phase was obtained. We evaluated all trees in a likelihood framework by computing likelihood scores for all trees using the LG matrix in PHYML v3.0 [105] with the following command (phyml -i 01-Input.phy -c 4 -m LG -a e -o lr -f e -u 01-Input.tre -v e -d aa -b 0 -s NNI). We then chose the tree with the highest likelihood from all 50 maximum likelihood searches and both Bayesian trees and applied both maximum likelihood and Bayesian consensus support values to the most likely tree, which was arranged and visualized using FigTree v1.3.1 [106]. Trees were rerooted in FigTree if needed, and then annotated manually using Adobe Illustrator. Nodes with support values between 50% and 100% were labeled on the trees. To explore consensus results among all of the result trees generated, we also computed 50% majority rule consensus trees with RAxML (-J MR option). We used PhyUtility [107] to examine the branch attachment frequency and leaf stability of MleiOpsin3. To examine the effect of MleiOpsin3 on the phylogeny, we removed this sequence and repeated the analysis.

## *Mnemiopsis *photoprotein protein expression and characterization

### PCR and expression cloning

Adult *Mnemiopsis *specimens were collected during Spring 2011 from the Atlantic Coast off the pier at the Rosenstiel School of Marine and Atmospheric Science in Miami, FL, USA using dip nets and then stored in ethanol. Genomic DNA was extracted from tissue using DNeasy Blood and Tissue kit (Qiagen, Valencia, CA, USA) as per instructions. PCR primers for subcloning are detailed in Additional file 14. Genomic DNA served as the template for PCR using Phusion DNA Polymerase (New England BioLabs, Inc., Ipswich, MA, USA), and cycling conditions were as follows: initial denaturation step 98°C for 30 s, then 35 cycles of 98°C for 10 s, 65°C for 30 s, 72°C for 30 s, and final extension of 72°C for 10 min. PCR products were purified and then incubated at 72°C for 12 min for 3'-adenine residue addition and subcloned into the StrataClone PCR cloning vector pSC-A-amp/kan (Agilent Technologies, Santa Clara, CA, USA). Sanger sequencing was used to identify full-length clones for expression cloning. Expression cloning primers are detailed in Additional file 14.

Purified mini-prep DNA from PCR cloning was used as a template for expression cloning using Phusion DNA Polymerase, and cycling conditions were as follows: initial denaturation step 98°C for 30 s, then 35 cycles of 98°C for 10 s, 65°C for 30 s, 72°C for 30 s, and final extension of 72°C for 10 min. Purified product was digested with restriction enzymes (New England BioLabs, Inc.) and ligated into a pCold expression vector (Clontech Laboratories, Inc.) using Invitrogen T4 DNA ligase (Life Technologies, Grand Island, NY, USA) containing 8-histidine residues and maltose binding protein on the N-terminus; MleiPP9 was ligated into the same expression vector but without the maltose binding protein tag.

### Expression and purification from *Escherichia coli *cells

Sanger sequencing was used to confirm full-length in-frame clones that were transformed into *Escherichia coli *strain BL21 (DE3; New England BioLabs, Inc.) by electroporation. Single colonies were picked into 5-mL 2XYT media containing 100 μg/mL carbenicillin (Fisher Scientific, Pittsburgh, PA, USA) and grown at 37°C overnight with shaking at 250 rpm. The resulting culture was then used to inoculate 500 mL of 2XYT and antibiotics. Once the optical density at 600 nm reached between 0.5 and 0.6, cultures were placed on ice for 60 min. To this was added 0.8 mM of isopropyl-β-D-thiogalactopyranoside (Fisher Scientific) and the cultures were grown for at least 24 h at 15°C while shaking. Cell pellets were harvested by centrifugation at 4,000 ×g for 30 min. Cells were lysed on ice for 30 min in buffer containing 1 mg/mL lysozyme, sonicated, and pelleted by centrifugation. The remaining supernatant was applied to nitrilotriacetic acid beads (Qiagen) at 4°C for 1 h with gentle agitation. To elute recombinant protein, 2 mL of elution buffer containing 250 mM imidazole was applied to the columns and fractions were collected, dialyzed using spin filters with a membrane molecular weight cut-off of 10,000 (EMD Millipore, Billerica, MA, USA), and digested with tobacco etch virus protease overnight at 4°C. This reaction was again purified using nitrilotriacetic acid beads (Qiagen) and recombinant protein was collected as the flow-through.

### Photoprotein analysis

Each step of purification was subjected to protein gel electrophoresis following the Laemmli method (data not shown). SDS sample buffer (Life Technologies) was added to 1 to 5 µL of each sample to a total volume of 10 µL and loaded on a 4% to 20% Tris-Glycine PAGE gel (Life Technologies) with 8 µL of BenchMark Prestained Protein Ladder (New England BioLabs, Inc.). Gels were run according to manufacturer's specifications. Coomassie Blue staining (Life Technologies) was used to visualize gel bands.

### Photoprotein spectral analysis

The following conditions were used for luminescence assays unless otherwise noted: purified tobacco etch virus-digested apo-photoprotein was added to 50 mM phosphate buffer with 450 mM NaCl, 5 mM Ambion EDTA (Life Technologies) and 1 mg/mL coelenterazine in dimethyl sulfoxide (Fisher Scientific) and regenerated for longer than 16 h without light at 4°C. The pH of this buffer was adjusted to 8.0, 9.0, or 10.0. Luminescence spectra were measured using a Roper Scientific black-illuminated CCD camera mounted to an Acton series SpectraPro monochromator (Princeton Instruments, Princeton, NJ, USA). Emission spectra were collected using WinSpec software and exported to R, where data were normalized and graphed using a spline curve-fit analysis.

### *Mnemiopsis *opsin protein expression and characterization

The full-length *MleiOpsin2 *cDNA was modified by RT-PCR using forward (5'-NNNNGAATTCCACCATGTCAAGCCCCAACG-3') and reverse (5'-TATAGTCGACAGTCGGCCTCCAAAGTAAAGG-3') primers. This cDNA, containing *Eco*RI, Kosak, and *Sal*I sequences, was cloned into the *Eco*RI and *Sal*I restriction sites of the expression vector pMT5. The plasmid was expressed in COS1 cells by transient transfection. MleiOpsin2 pigment was regenerated by incubating the opsin with 11-*cis*-retinal (Storm Eye Institute, Medical University of South Carolina, Charleston, SC, USA) and was purified using immobilized 1D4 (The Culture Center, Minneapolis, MN, USA) in buffer W1 (50 mM N-(2-hydroxyethyl) piperazine-N'-2-ethanesulfonic acid, pH 6.6, 140 mM NaCl, 3 mM MgCl_2_, 20% (w/v) glycerol, and 0.1% dodecyl maltoside) [108]. UV visible spectrum was recorded at 20°C using a Hitachi U-3000 dual beam spectrophotometer (Mountain View, CA, USA). The data were analyzed using Sigmaplot software (Jandel Scientific, San Rafael, CA, USA).

### *Mnemiopsis *photoprotein and opsin mRNA expression

Genes of interest were isolated using RACE-PCR (Clontech Laboratories, Inc.) (see Confirmation of individual *Mnemiopsis *photoprotein sequences for details). *In situ *hybridizations (probes designed for photoproteins from each sequence group: *MleiPP1, MleiPP7, MleiPP8*; plus opsins *MleiOpsin1 *and *MleiOpsin2*) were performed as previously described [67]. Full- or partial-length sequences, ranging in size from 600 to 2000 bp, were used to transcribe digoxigenin-labeled RNA probes. We detected these probes using an alkaline phosphatase-conjugated digoxigenin antibody, utilizing the substrates nitro blue tetrazolium (NBT) and 5-Bromo-4-chloro-3-indolyl phosphate (BCIP) to then detect the alkaline phosphatase activity (Roche Applied Science, Indianapolis, IN, USA). Specimens were mounted in 70% glycerol, viewed under a Zeiss AxioSkop, and imaged using an AxioCam (Thornwood, NY, USA).

Co-fluorescent *in situ *hybridizations of one opsin gene (*MleiOpsin2*) and one photoprotein gene (*MleiPP1*) were based on *Xenopus *protocols available at Xenbase [109]. A fluorescein-labeled photoprotein probe was mixed with a digoxigenin-labeled opsin probe (*MleiOpsin2*), and hybridization was performed as before. Following hybridization and washes, the opsin probe was detected first using an anti-digoxigenin-alkaline phosphatase antibody (Roche Applied Science) and NBT/BCIP substrates. When the signal was sufficiently developed, embryos were washed five times with PBS. Endogenous peroxidase was then quenched via a 30 min wash in 1% hydrogen peroxide. The photoprotein probe was then detected using an anti-fluorescein-peroxidase antibody (Roche Applied Science). Following washes in PBS, embryos were incubated in tyramide-fluorescein isothiocyanate plus 0.001% hydrogen peroxide for 45 minutes. If needed, this tyramide step was repeated up to three times. Embryos were then washed with PBS until the signal was sufficiently visible. Embryos were incubated with Hoechst 33432 for 30 min to visualize nuclei, and then washed twice more in PBS.

Opsin and photoprotein fluorescent *in situ *hybridizations were imaged using a Zeiss LSM710 confocal microscope. The opsin NBT/BCIP visualized staining was imaged using reflective confocal microscopy [110]. Individual stacks were merged into a three-dimensional projection using the Zen software (Carl Zeiss Microscopy, Thornwood, NY, USA).

### *Nematostella *photoprotein-like mRNA expression

The two *Nematostella *photoprotein-like genes were isolated via 3'-RACE-PCR using mixed-stage (0 to 7 days post-fertilization) *Nematostella *cDNA. These clones were used to generate digoxigenin-labeled RNA probes for *in situ *hybridization. These probes were 500 bp (*NvecPP1*) and 1,200 bp (*NvecPP2*). *Nematostella *embryos and polyps were fixed in 3.7% formaldehyde and 0.2% glutaraldehyde as previously described [111]. *In situ *hybridizations were performed at 65°C, as previously described [111,112]. Specimens were mounted in 70% glycerol and imaged on an AxioSkop2 with an AxioCam (Carl Zeiss Microscopy).

## Abbreviations

7TM: seven-transmembrane; BCIP: 5-Bromo-4-chloro-3-indolyl phosphate; BLAST: Basic Local Alignment Search Tool; bp: base pair; EST: expressed sequence tag; GFP: green fluorescent protein; Go/RGR: Go-coupled plus retinochrome, retinal G protein-coupled receptor; NBT: nitro blue tetrazolium; NCBI: National Center for Biotechnology Information; ORF: open reading frame; PBS: phosphate-buffered saline; PCR: polymerase chain reaction; RACE: rapid amplification of cDNA ends; RT: reverse transcriptase; UTR: untranslated region.

## Competing interests

The authors declare that they have no competing interests.

## Authors' contributions

CES, KP, MLP, AMR, JFR, SY, SHDH, MQM, and ADB conceived and designed the experiments. CES, KP, MLP, AMR, DS, and TT performed the experiments. CES, KP, MLP, AMR, JFR, MP, JG, SYB, RWB, SY, SHDH, MQM and ADB analyzed the data. RWB, SY, SHDH, MQM, and ADB contributed reagents, materials, or analysis tools. CES, KP, MLP, AMR, JFR, SHDH, MQM, and ADB wrote the paper. All authors read and approved the final manuscript.

## Supplementary Material

Additional file 1 **Overall pairwise photoprotein percent amino acid identity comparisons for a subset of photoproteins and photoprotein-like sequences**. Click here for file

Additional file 2 **Predicted molecular weight (kDa) and isoelectric point (pH) values and averages for the 10 *Mnemiopsis *photoproteins**. Click here for file

Additional file 3 **Global analysis results for sequence pairs with evidence for recombination from GENECONV for the *Mnemiopsis *photoproteins**. Click here for file

Additional file 4 **Alignment of EF-hand domains I, III, and IV of select photoprotein and photoprotein-like sequences**. Important calcium ligand residues in the 12-residue calcium binding loops within each EF-hand domain are indicated with black triangles. Columns of residues are shaded as in Figure 2. Species are abbreviated as follows: Ac = *Aequorea coerulescens*; Aque = *Amphimedon queenslandica*; Cg = *Clytia gregarium*; Mc = *Mitrocoma cellularia*; Mlei = *Mnemiopsis leidyi*; Nvec = *Nematostella vectensis*; Og = *Obelia geniculata*. Click here for file

Additional file 5 ***In situ *hybridizations showing mRNA expression patterns for two photoprotein-like genes from *Nematostella***. **(A) ***NvecPP2*: Panels A-C and E-H are lateral views, with the oral pole to the left. Panel D is an oral view. Expression is first detected in the early polyp stage (A-D) in the endoderm, particularly in the mesenteries. There is also an additional expression in the apical tuft (B). In older polyp stages (E-H), the expression in the mesenteries decreases, while the apical tuft expression remains. There is also an additional expression domain in the tips of newly forming tentacles (F, H). **(B) ***NvecPP1*: Panels A-C and E-H are lateral views, with the oral pole to the left and Panel D is an oral view. Expression of *NvecPP1 *is detected in the late planula stages (A), in small patches in the endoderm towards the oral pole. In early polyp stages (B-D), the expression continues and forms a ring in the endoderm, although expression is highest in the areas between where the tentacles grow. In older polyp stages (E-H), the endodermal expression slowly decreases. In these stages, there is also endodermal expression in the tips of newly forming tentacles (F-H). Format: TIF Click here for file

Additional file 6 **Opsin seven-transmembrane pairwise comparisons of percent amino acid identity (top right) and similarity (bottom left) for a subset of opsin sequences**. Species are abbreviated as follows: Hs = *Homo sapiens*; Nv = *Nematostella vectensis*; Mlei = *Mnemiopsis leidyi*; Ppil = *Pleurobrachia pileus*. Click here for file

Additional file 7 **Alignment of the seven-transmembrane region of select opsin sequences**. Columns of residues are shaded by similarity group conservation (defined by GeneDoc and the BLOSUM62 matrix) where black shows 100%, dark grey shows ≥80%, and light grey shows ≥60% similar residues in a column. Gene name abbreviations: MWS = medium-wavelength sensitive; RGR = retinal G protein-coupled receptor. Conserved sites of chromophore linkage at Lys296 (black circle), disulfide bridge formation at Cys110-Cys187 (black stars) and signal propagation at Glu134-Arg35-Tyr136 (black square) are indicated. Sites corresponding to the acidic counterion in the vertebrate pigments (Glu113) and the counterion in other species (Glu181) are also indicated (black triangles). Species are abbreviated as follows: Dm = *Drosophila melanogaster*; Es = *Euprymna scolopes*; Hs = *Homo sapiens*; Mlei = *Mnemiopsis leidyi*; Nv = *Nematostella vectensis*. Click here for file

Additional file 8 **Fifty percent majority rule consensus tree of photoprotein genes**. Click here for file

Additional file 9 **Fifty percent majority rule consensus tree of opsin genes**. Click here for file

Additional file 10 **Fifty percent majority rule consensus tree of opsin genes minus MleiOpsin3**. Click here for file

Additional file 11 **RACE primers used for 5'- and 3'-RACE-PCR confirmation of *Mnemiopsis *photoprotein genes**. Click here for file

Additional file 12 **Photoprotein multiple sequence alignment in FASTA format**. Species abbreviations as in Figure 3. Click here for file

Additional file 13 **Opsin seven-transmembrane multiple sequence alignment in FASTA format**. Click here for file

Additional file 14 **Primers used for cloning a subset of *Mnemiopsis *photoproteins for expression experiments**. A first set of PCR primers was used to clone the full-length proteins; a second set of expression cloning primers was used to add restriction sites for cloning proteins into an expression vector. For = forward; Rev = reverse. Click here for file
