# Supplementary figures and images for: Genomic organization, evolution, and expression of photoprotein and opsin genes in Mnemiopsis leidyi: a new view of ctenophore photocytes
Source: BMC Biol. 2012 Dec 21;10:107. doi: 10.1186/1741-7007-10-107 (PMC3570280; doi:10.1186/1741-7007-10-107)

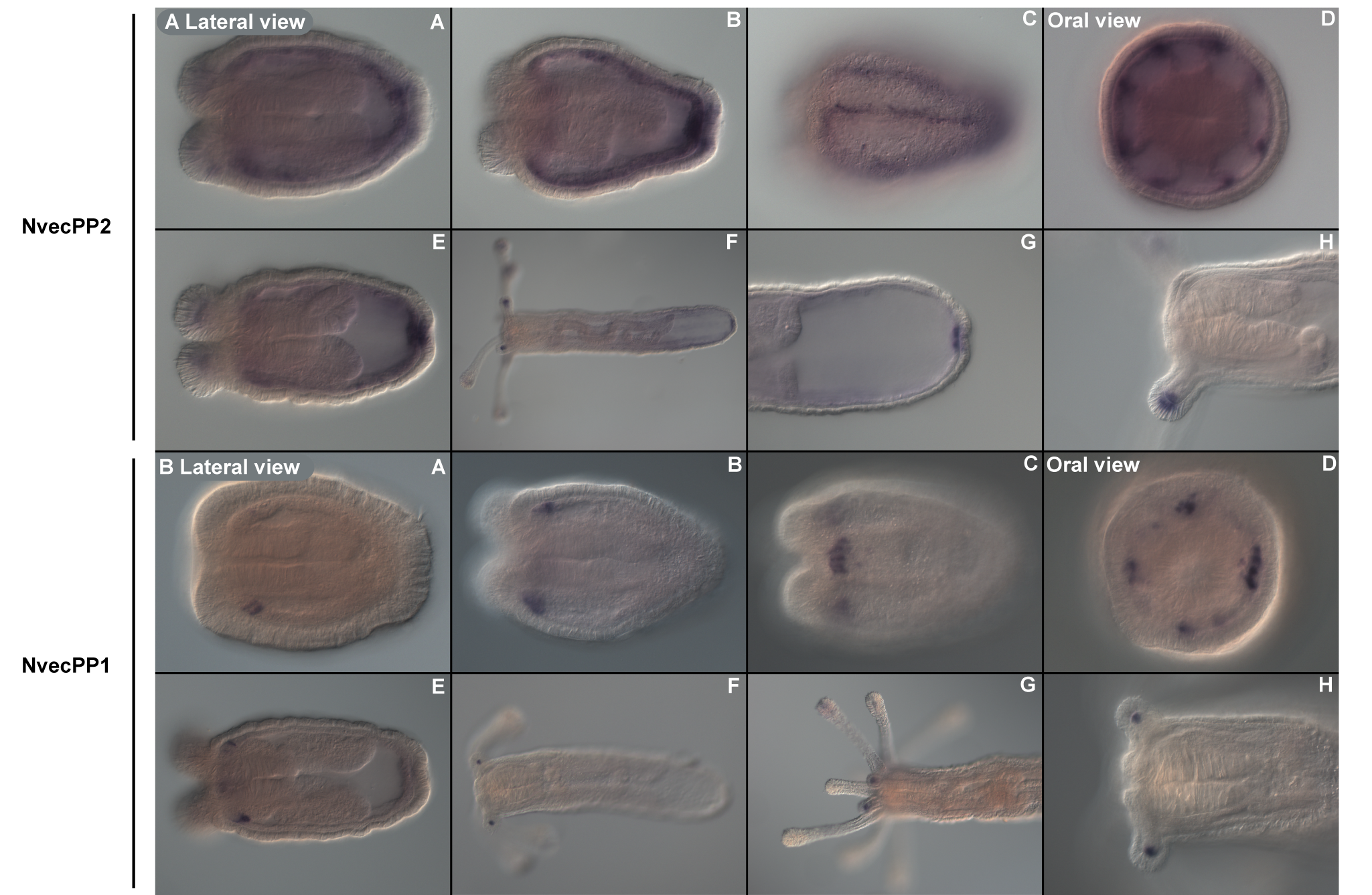

Supplement: Additional file 5 — In situ hybridizations showing mRNA expression patterns for two photoprotein-like genes from Nematostella. (A) NvecPP2: Panels A-C and E-H are lateral views, with the oral pole to the left. Panel D is an oral view. Expression is first detected in the early polyp stage (A-D) in the endoderm, particularly in the mesenteries. There is also an additional expression in the apical tuft (B). In older polyp stages (E-H), the expression in the mesenteries decreases, while the apical tuft expression remains. There is also an additional expression domain in the tips of newly forming tentacles (F, H). (B) NvecPP1: Panels A-C and E-H are lateral views, with the oral pole to the left and Panel D is an oral view. Expression of NvecPP1 is detected in the late planula stages (A), in small patches in the endoderm towards the oral pole. In early polyp stages (B-D), the expression continues and forms a ring in the endoderm, although expression is highest in the areas between where the tentacles grow. In older polyp stages (E-H), the endodermal expression slowly decreases. In these stages, there is also endodermal expression in the tips of newly forming tentacles (F-H). Format: TIF [file 1741-7007-10-107-S5.TIFF]
